# Endothelial β-PIX (ARHGEF7) supports actomyosin mediated expulsion of VWF through dynamic reorganization of the cytoskeleton

**DOI:** 10.1091/mbc.E25-10-0483

**Published:** 2026-05-04

**Authors:** Sammy El-Mansi, Karishma M. Deegan, Thomas D. Nightingale

**Affiliations:** ^a^Centre for Microvascular Research, William Harvey Research Institute, Barts and the London School of Medicine and Dentistry, Queen Mary University of London, London EC1M 6BQ; University of Queensland

## Abstract

Endothelial cells promote thrombosis and hemostasis through the secretion of von Willebrand Factor (VWF) from their secretory granules—the Weibel-Palade bodies (WPBs). In response to specific stimuli, dynamic actin nucleation and remodeling of the cytoskeleton facilitates the expulsion of ultra-large VWF multimers to elevate plasma VWF and to form a platform for platelet capture and thrombus formation. P21 activated kinase 2 (PAK2) is a crucial regulator of the actin cytoskeleton and is essential for VWF secretion in response to secretagogues which utilize actomyosin mediated exocytosis. Here, we characterize the role of β-PAK-interacting exchange factor (β-PIX) in WPB exocytosis. Inhibition of β-PIX function prevents dynamic cytoskeletal remodeling resulting in reduced VWF secretion. Depletion of β-PIX using siRNA reduced the number of WPB fusion events, prolonged the time taken for GFP-VWF to be secreted post-fusion and delayed kinetics of the exocytic actomyosin ring. Use of full length and truncated β-PIX demonstrated that the PAK interacting and GEF domain mediate cytoskeletal remodeling whereas only the full-length construct could rescue VWF secretion. β-PIX regulates both septin ring formation and cofilin mediated actin remodeling during actomyosin ring function. These data identify β-PIX as a regulator of endothelial exocytosis through supporting actomyosin-mediated expulsion of VWF.

## Abstract (executive summary)

- An actomyosin ring assembles around fused endothelial storage organelles to expedite exocytosis by providing motive force. Although several components of this ring have been characterized, its therapeutic targeting remains unclear.
- Using biochemical assays and live-cell imaging, we detail a role for β-PIX (ARHGEF7) in cytoskeletal remodeling. Inhibition of β-PIX results in delayed release of VWF and an increase in ring lifetime. Using knockdown and rescue experiments with mutated β-PIX, we define the domains essential for function.
- This represents the first characterization of β-PIX function in actin coat remodeling. Controlling β-PIX function represents a novel means for limiting VWF release.

## INTRODUCTION

Endothelial cells rapidly respond to blood vessel injury or infection by releasing hemostatic and inflammatory proteins from their secretory granules called Weibel-Palade Bodies (WPBs). Upon stimulation, WPBs traffic to the cell surface to secrete cargo such as von Willebrand factor (VWF), essential for platelet recruitment, and P-selectin, which facilitates the capture and subsequent rolling of immune cells. This process underpins the endothelium's key role in the control of inflammation and blood clotting (McCormack *et al.*, 2017).

Like other secretory granules, WPB exocytosis depends on efficient trafficking to the plasma membrane (PM), fusion, and expulsion of contents. After their biogenesis at the trans-Golgi network, long-range microtubule transport delivers WPB to the cell periphery (Manneville *et al.*, 2003). In addition to F-actin supporting short-range movement (Manneville *et al.*, 2003), it acts as an anchor to facilitate maturation—a process driven by a complex of MyoVa/MYRIP/Rab27a (Nightingale *et al.*, 2009; Conte *et al.*, 2016). A meshwork of cortical actin acts as a barrier to exocytosis, and its dynamic reorganization is required for effective exocytosis (Nightingale *et al.*, 2011; Nightingale *et al.*, 2012; Han *et al.*, 2017; Li *et al.*, 2018; Nightingale *et al.*, 2018; Mietkowska *et al.*, 2019; McCormack *et al.*, 2020). Following fusion with the PM, actin and myosins rapidly assemble into a transient ring or coat that persists for approximately 20 s before contraction coincides with the expulsion of VWF. This force-generating process is essential for the efficient release of ultra-large, unwieldy VWF multimers in response to stimulation by specific cAMP-raising and PKC-activating secretagogues (forskolin, epinephrine, VEGF, and PMA) (Nightingale *et al.*, 2018).

It is also of note that rings/coats of actin are recruited preferentially at the longest and most pro-hemostatic WPBs in the cells (McCormack *et al.*, 2020), further emphasizing the need for additional machinery to squeeze ultralarge multimers out of the cell. Supporting this, similar actomyosin coats are observed in other systems, including *Drosophila* glue granules (Tran *et al.*, 2015; Merrifield, 2016; Rousso *et al.*, 2016), alveolar type II cell lamellar bodies (Miklavc *et al.*, 2012), and salivary gland acinar cells (Ebrahim *et al.*, 2019), a common feature of which is large or viscous cargo.

Recent studies have considerably advanced our understanding of the molecular mechanisms underlying the formation and functions of actomyosin rings and coats. Upon WPB-PM fusion, phosphatidylinositol 4,5-bisphosphate (PIP2) is generated or transferred to the WPB membrane (Nguyen *et al.*, 2020). Amongst other functions, PIP2 serves as a docking site for the unconventional myosin-1c (Myo1c) through its plekstrin homology domain, thereby establishing a molecular link between the membrane and actin (El-Mansi *et al.*, 2024). Local polymerization of actin then follows in a manner dependent on RhoA (Mietkowska *et al.*, 2019) and Spire1 (Holthenrich *et al.*, 2022).

Following initial actin polymerization, recruitment, and utilization of nonmuscle myosin isoforms is thought to occur in a temporally coordinated manner, coinciding with the recruitment of septin hetero-oligomers (El-Mansi *et al.*, 2023). Septin ring formation is dependent on p21-activated kinase 2 (PAK2), a serine/threonine kinase best known for orchestrating cytoskeletal remodeling downstream of Rho GTPases (Cdc42/Rac). Inhibition of septins delays the contraction kinetics of the actomyosin ring and reduces VWF secretion. These findings support a working hypothesis that septins regulate the mechanics of actomyosin rings and coats, ensuring VWF secretion.

Inhibition of signaling pathways that govern cytoskeletal remodeling and actomyosin ring formation represents a practical therapeutic strategy for controlling WPB content release. In previous work, we demonstrated that pharmacological blockade of PAK activity—either at the Cdc42/Rac binding domain (IPA-3) or at the catalytic kinase domain (FRAX486)—significantly reduced VWF secretion in vitro and in vivo. Therefore, we propose that the PAK2 signaling pathway is an unexploited therapeutic target for thrombotic disease. However, it's targeting presents substantial challenges (Chen *et al.*, 2025). First, the allosteric inhibitor IPA-3 is not suitable for use physiologically due to an unstable disulfide bond under reducing conditions (Kim *et al.*, 2016), and secondly, ATP-competitive inhibitors of PAKs are seldom selective due to the presence of six closely related PAK isoforms. To date, only one selective PAK (PAK4) inhibitor has reached clinical investigation (Pfizer anticancer drug PF309; Mileshkin *et al.*, 2011). Thus, alternative approaches to target this important signaling pathway are of significant interest.

We focused on alternative pressure points within this vital signaling cascade. The guanine nucleotide exchange factor β-PIX (ARHGEF7) is a key activator of Cdc42/Rac and can also bind PAK2 directly via its SH3 domain (Bagrodia *et al.*, 1998; Manser *et al.*, 1998; ten Klooster *et al.*, 2006). Using siRNA depletion and expression of mutant constructs alongside biochemical assays and high-resolution imaging, we demonstrate a role for β-PIX in supporting actomyosin ring function. Collectively, these findings identify β-PIX as a previously unrecognized regulator of VWF secretion in endothelial cells.

## RESULTS

### Loss of β-PIX prevents dynamic cytoskeletal remodeling in response to phorbol ester

As a guanine nucleotide exchange factor for Cdc42/Rac, we first focused our analysis on the cytoskeletal phenotype of control and β-PIX-depleted cells in the presence and absence of PMA. PMA is a cell-permeable activator of protein kinase C, which rapidly induces changes to the cytoskeleton, such as membrane ruffling downstream of Rac (Ridley *et al.*, 1992). As it surpasses membrane-bound receptors, it circumvents possible confounding effects of β-PIX function on receptor trafficking and endocytosis. Additionally, PMA robustly promotes the recruitment of actomyosin rings during WPB exocytosis and is therefore a useful tool to study their function (Nightingale *et al.*, 2011; Nightingale *et al.*, 2018; McCormack *et al.*, 2020).

**FIGURE 1:**
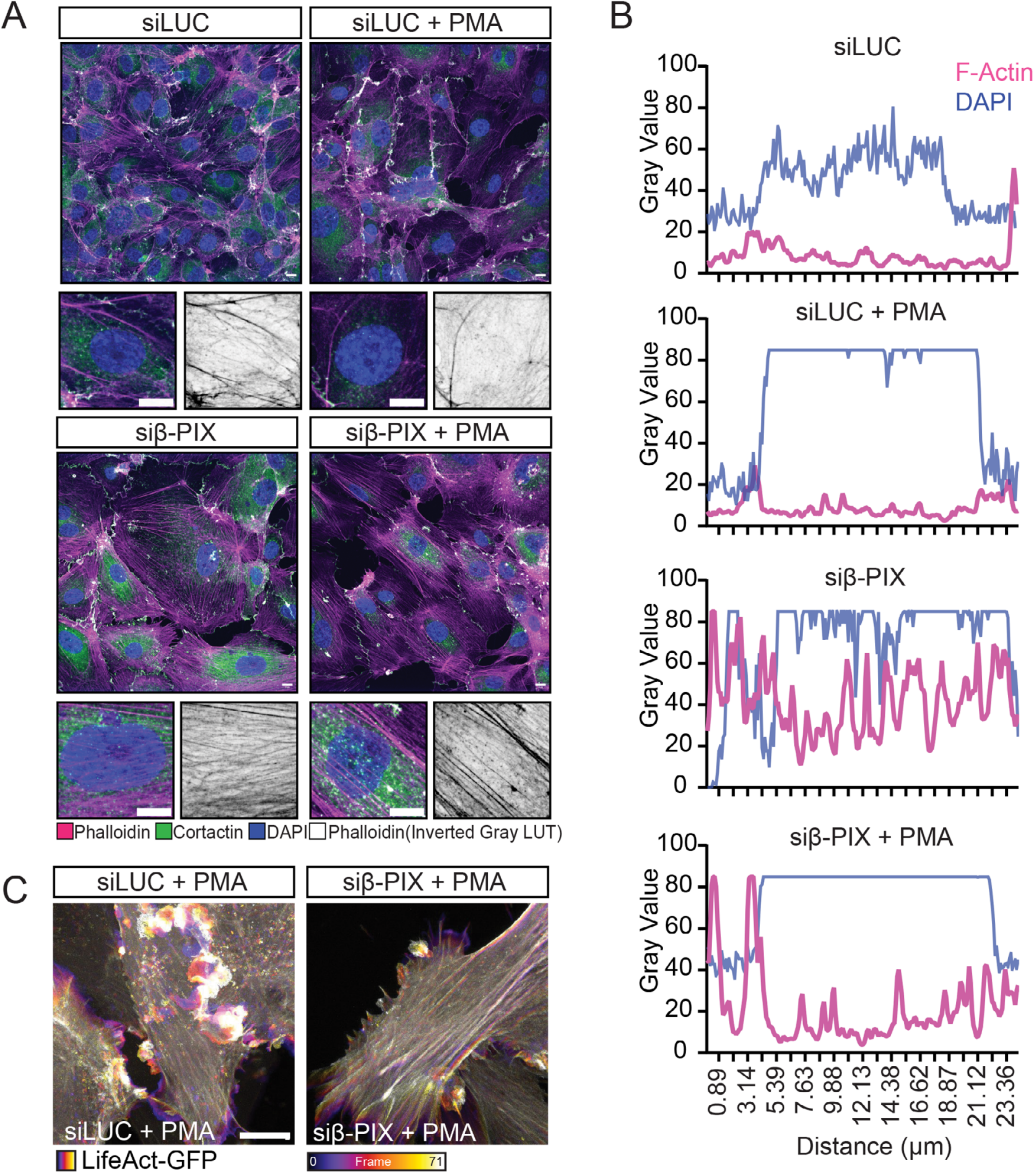
Endothelial β-PIX regulates actin cytoskeleton architecture. (A) Control (LUC) and β-PIX KD cells were left untreated or stimulated with PMA for 5 min before fixation, permeabilization, and IF staining. Visualization of F-Actin (magenta), cortactin (green), and DAPI (blue) by confocal microscopy highlighted the presence of actin ruffles in control cells treated with PMA but not β-PIX KD cells. Moreover, β-PIX KD cells displayed prominent dorsal F-Actin stress fibers under both conditions. (B) Line graphs represent F-Actin distribution over the nuclear region as determined by grey values. Undulating peaks represent stress fibers. (C) Temporal coded maximum intensity projects from live cell imaging (10-min acquisitions) of Lifeact-GFP expressing HUVEC, which show actin dynamics in real time following stimulation with PMA. Dynamic membrane ruffles (white) are only seen following stimulation in control cells (LUC). Scale bar 10 µm.

We determined a potent KD of endogenous β-PIX in siRNA-transfected HUVEC (Supplemental Figure S1), allowing the robust assessment of its effect in endothelial cells. Immunostaining for cortactin (a marker of membrane ruffles; Wu and Parsons, 1993) demonstrated the presence of membrane ruffles in PMA-stimulated HUVEC, which was abrogated in β-PIX KD cells. Through phalloidin staining, we determined a distinct phenotype in β-PIX-depleted cells, typified by prominent F-actin stress fibres (Figure 1A). To quantify, surface plots of F-actin signal over the nuclear region were plotted, where undulating peaks represent stress fibres (Zonderland *et al.*, 2019; Figure 1B). Live cell imaging of PMA-stimulated HUVEC expressing the actin probe, LifeAct-GFP (Riedl *et al.*, 2008), confirmed both phenotypes (Supplemental Movies S1 and S2). Shown here are temporal color-coded maximum intensity projections (Supplemental Figure 1C).

### PAK- and Cdc42/Rac-interacting domains are required for cytoskeletal remodeling

**FIGURE 2:**
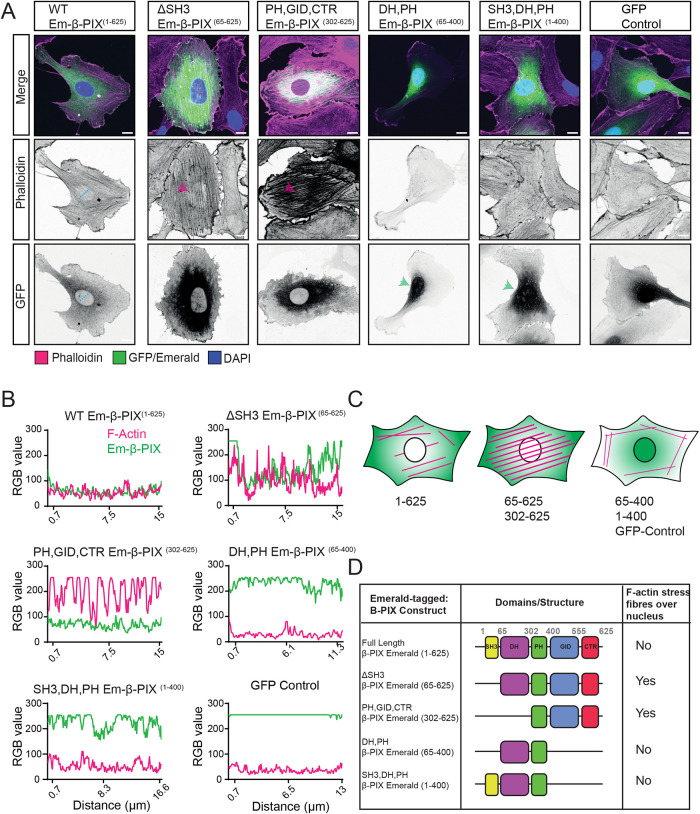
SH3 and DH domains of β-PIX regulate actin cytoskeleton architecture. (A) Overexpression of truncated mutants has dominant negative effects on cytoskeletal architecture. Confocal images of F-actin structure (magenta) and Emerald-GFP-β-PIX (Em-β-PIX: green) localization. Green arrows indicate mutant constructs lacking the GID and CTR translocate to the nucleus. Magenta arrows indicate dense F-Actin dorsal stress fibers. Note differences in neighboring cells that do not express the construct. The blue dashed line shows the region used to quantify the RGB surface plot. Scale bars are 10 µm. (B) Localization of Em-β-PIX and the F-actin architecture was assessed by plotting the RGB profile above the nucleus. (C) Schematic reflecting changes to cytoskeletal structure following transient overexpression of full-length and mutant Em-β-PIX constructs. (D) Schematic representation of the domains and structure of Em-β-PIX constructs and their effect on the cytoskeletal architecture.

To better understand how β-PIX regulates cytoskeletal remodeling, we utilized Emerald-GFP-tagged β-PIX truncated mutants (Em-β-PIX). β-PIX has five domains: an *N*-terminal SH3 domain (PAK-binding), a Dbl-homology domain (DH) with Rac1 and Cdc42 guanine nucleotide exchange factor (GEF) activity, a lipid-binding plekstrin homology domain (PH), a GIT1/2 Interacting Domain (GID), and a poorly understood *C*-terminal Region (CTR). Upon overexpression of these mutant constructions, we noticed two phenotypes. First, truncation of the GID and CTR resulted in nuclear localization of Em-β-PIX^(1-400&65-400)^ (Green arrows). Secondly, overexpression of mutant Em-β-PIX constructs lacking the SH3 or DH domain phenocopied the effect of β-PIX depletion on the cytoskeletal architecture (Magenta arrows: dense F-Actin peri-nuclear stress fibers; Figure 2A). HUVEC expressing Em-β-PIX^(1-400)^ or Em-β-PIX^(65-400)^ did not show dominant negative effects in this regard. Once more, plotting the surface profile of F-actin staining over the nuclear region confirmed these phenotypes (Figure 2, B–D). It was informative that overexpression of a GEF dead point mutant (L238R/L293S) recapitulated this cytoskeletal phenotype (Supplemental Figure S2).

### Loss of β-PIX impairs fusion and delays GFP-VWF expulsion

**FIGURE 3:**
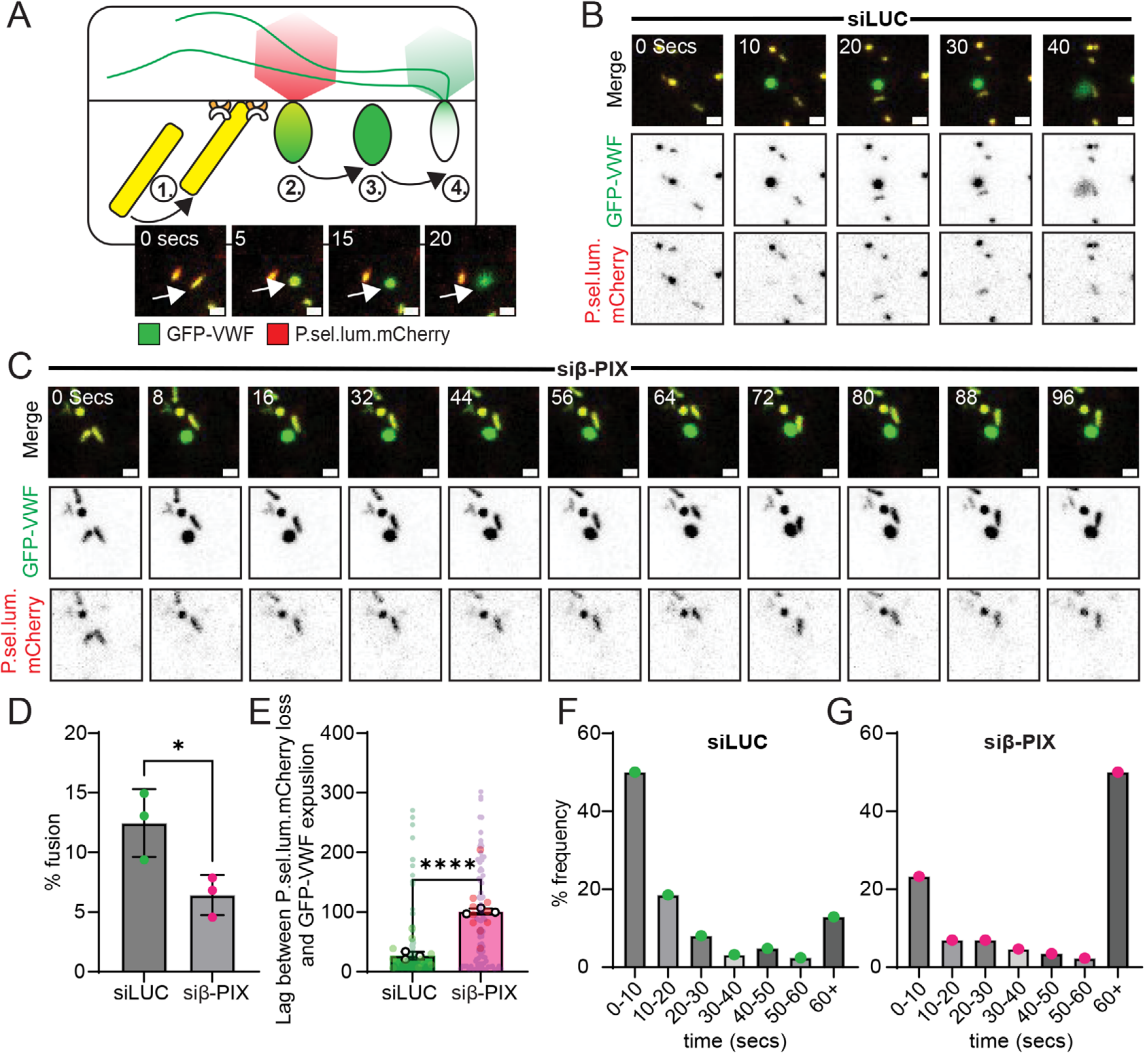
Depletion of endothelial β-PIX impedes WPB fusion and delays VWF expulsion post-fusion. (A) Schematic of the live cell imaging approach to study WPB fusion. HUVEC were transfected with plasmid DNA to transiently express GFP-VWF and a soluble marker lost upon fusion with the PM, an mCherry-tagged lumenal domain of P-selectin (P.sel.lum.mCherry). (B) Example still images from a movie that probes an individual WPB fusion event and expulsion of GFP-VWF in control cells and (C) β-PIX KD cells (PMA stimulation). (D) Quantification of WPB fusion events within 5 min in control (LUC) and β-PIX KD cells. A total of 22 cells were analyzed over three independent experiments. Students *t* test *P* < 0.05. (E) Mean duration of delay between loss of fusion marker and GFP-VWF expulsion. In total, 208 events were analyzed in 22 cells over three independent experiments. Students *t* test *P* < 0.001. (F) Distribution of frequency of events in Control (LUC) and (G) β-PIX KD cells.

Having determined that the inhibition of β-PIX function alters cytoskeletal architecture, we next assessed the effect on Weibel-Palade body exocytosis and VWF secretion. Utilizing live cell confocal imaging of HUVEC expressing GFP-VWF and a soluble fusion marker, P-selectin luminal domain mCherry (P.sel.lum.mCherry), we performed quantitative analysis of WPB fusion events (Figure 3A). Following stimulation with PMA, WPBs that fuse with the PM collapse, changing from rod-shaped to spherical. Accompanying this morphological change, P.sel.lum.mCherry is lost, whereas GFP-VWF expulsion is delayed by approximately 20 s. Representative examples of WPB fusion events in control (Figure 3B) and β-PIX-depleted cells (Figure 3C). First, PMA stimulation led to ∼12.5% of WPB fusion over a period of 5 min. This response was reduced to ∼6.4% in β-PIX-depleted cells (Students *t* test *p* < 0.05; Figure 3D). The time delay (lag time) between loss of P.sel.lum.mCherry and loss of GFP-VWF following fusion and collapse of the WPB indicates the efficiency of VWF expulsion. Actomyosin rings aid this process, and inhibitors of actin and myosin I/II (e.g., Cytochalasin, Jasplakinolide, Blebbistatin, and Pentachloropseudilin) all delay GFP-VWF release post-fusion (Nightingale *et al.*, 2011; El-Mansi *et al.*, 2024). Here, β-PIX KD significantly extended the time between loss of P.sel.lum.mCherry and the disappearance of spherical GFP-VWF signal (27 ± 5 secs vs 101.3 ± 4secs, *p* < 0.0001; Figure 3E). This was reflected by a shift in the distributions of frequencies of events lasting less than 10 s and more than 60 s. (Figure 3F and G).

### Loss of β-PIX delays actin dynamics during exocytosis

**FIGURE 4:**
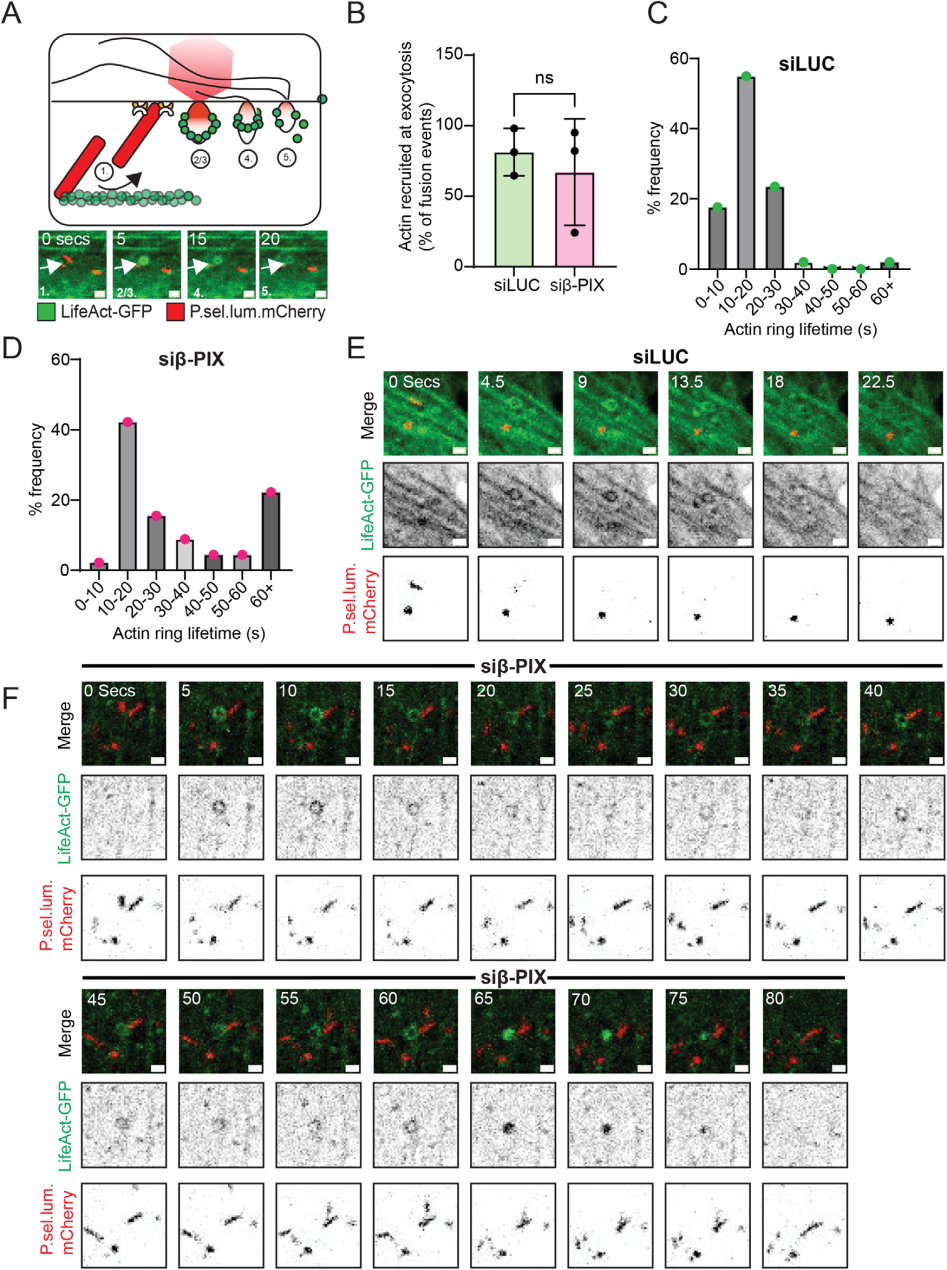
Depletion of endothelial β-PIX influences WPB exocytosis through impeding cytoskeletal remodeling (A) HUVEC were co-transfected with LifeAct-GFP and P.sel.lum.mCherry to image actin dynamics during exocytosis. (B) Quantification of the percentage of fusion events that recruit actin filaments during exocytosis. Distribution of frequency of the duration of LifeAct-GFP actin rings in (C) LUC and (D) β-PIX (96 events in 25 cells over three experiments). (E) Stills from live cell imaging videos of actin dynamics during WPB fusion events in LUC (F) β-PIX-depleted cells.

Following fusion and pH-driven collapse of the WPB, F-actin, nonmuscle myosins, and unconventional myosins are recruited in a ring structure, which aids the expulsion of VWF (Nightingale *et al.*, 2011; El-Mansi *et al.*, 2024). To investigate whether β-PIX supports this machinery, we co-transfected HUVEC with P.sel.lum.mCherry and LifeAct-GFP and performed live cell imaging (Figure 4A). We first quantified the proportion of WPB fusion events that recruited actin rings during exocytosis (Figure 4B). Depletion of β-PIX did not change the number of events that recruited actin, suggesting β-PIX is not centrally involved in nucleation events. However, β-PIX-depleted cells displayed longer-lasting actin rings, consistent with delayed expulsion (Figure 4, C–F). These kinetics suggest delayed contraction/disassembly kinetics indicative of impaired function. Thus, β-PIX function is required for both pre and post-fusion roles in VWF secretion.

### Inhibition of β-PIX function reduces VWF secretion in response to secretagogues

**FIGURE 5:**
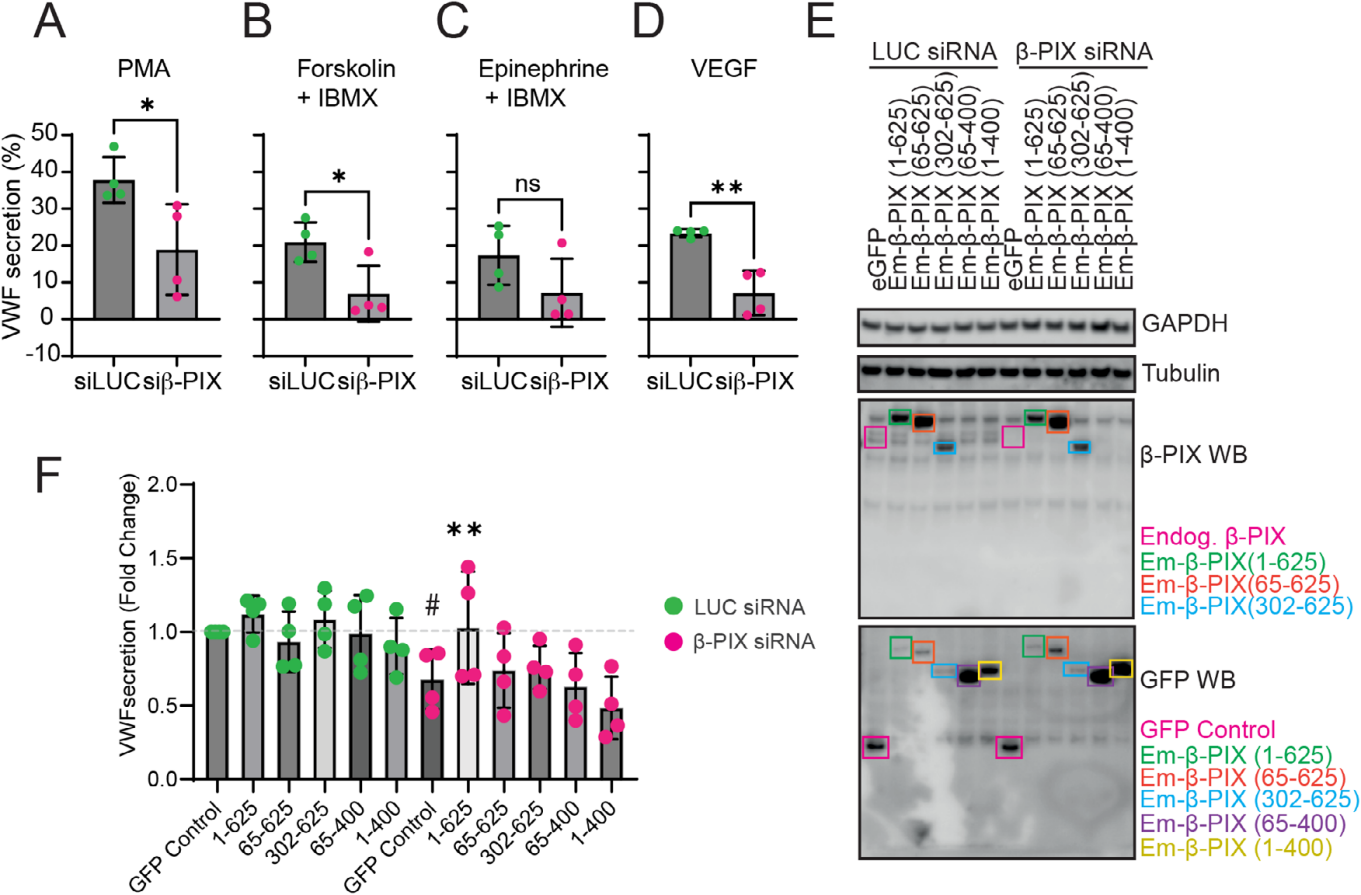
Endothelial β-PIX depends on its complete set of functional domains for VWF secretion (A–D) Luciferase (LUC) control or β-PIX KD HUVEC were exposed to (A) PMA (100 ng/ml), (B) Forskolin (10 µM and IBMX (100 µM), (C) Epinephrine (100 µM) and IBMX, or (D) VEGF (40 ng/ml) for 30 min before the amount of VWF secreted into the culture media was quantified using Near-infrared fluorescent dot blot. *N* = 4. (E) KD and rescue experiments were performed by transfecting LUC and β-PIX KD HUVEC with 5 µg plasmid construct before assaying 24 h later. Western blotting was used to detect target protein depletion (Magenta box - endogenous β-PIX) and construct expression using anti-β-PIX. A GFP antibody was used to detect the constructs lacking the C-terminal epitope (Purple and Yellow boxes). (F) KD and rescue HUVEC were stimulated with PMA (100 ng/ml), and VWF secretion was quantified through NIR dot blot. Only the full-length Em-β-PIX construct restored PMA-induced VWF secretion in β-PIX-depleted cells. Data are presented as a fold change. Ratio paired *t* test. *p < 0.05 **p < 0.01.

We next determined the effect of delayed fusion and actin ring impairment on VWF exocytosis. Using siRNA, endogenous β-PIX was depleted, and regulated VWF secretion was determined by secretion assay and NIR fluorescent dot blot (El-Mansi *et al.*, 2023) following exposure to a variety of different stimuli (Figure 5, A–D). β-PIX KD cells exhibited significantly reduced VWF secretion following exposure to PMA (37 ± 3.1% vs 18.9 ± 6.5%; Figure 5A), Forskolin (20.9 ± 2.7% vs 6.9 ± 3.8%; Figure 5B), and VEGF(23 ± 0.5% vs 7.1 ± 3%; Figure 5D). There was some evidence of an effect on epinephrine-induced release; however, this failed to reach significance (17.3 ± 3.4% vs 7.2 ± 4.6%; Figure 5C). Using a KD and rescue experiment, we assessed the effect of each of β-PIX's functional domains on VWF secretion. Western immunoblotting determined efficient KD of endogenous β-PIX (Magenta boxes) and expression of the full-length Em-β-PIX (1-625) as well as the truncated mutants in both LUC and β-PIX KD cells. (Figure 5E) The β-PIX antibody used was raised against a *C*-terminal located epitope, hence anti-GFP antibodies were required to detect Em-β-PIX (65-400) and (1-400; Figure 5E-lower panel). Only overexpression of the full-length Em-β-PIX was sufficient to rescue the defect in regulated VWF secretion (Ratio paired *t* test, *p < 0.05; Figure 5F). Demonstrating a marked difference from the stress fiber actin phenotype and indicating a requirement for other domains to target the exocytic actin ring.

### Downstream effects of β-PIX depletion

We previously demonstrated that the serine/threonine kinase, PAK2, regulates VWF secretion by controlling the formation of a septin ring that is necessary for actin ring contractility (El-Mansi *et al.*, 2023). As PAK2 is regulated by β-PIX (Manser *et al.*, 1998; Liu *et al.*, 2007; Reddy *et al.*, 2016; Zhou *et al.*, 2016), we determined the effect of β-PIX depletion on septin function (Supplemental Figure S3). β-PIX KD does not affect overall septin levels (Supplemental Figure S3, A and B) and results in a subtle difference in gross septin structure, with septin filaments enriched at perinuclear F-actin stress fibers (Supplemental Figure S3, C and D). Stimulated β-PIX KD cells exhibit a 53 ± 10.6% reduction in recruited exocytic septin rings (Supplemental Figure S3, E and F), similar in magnitude to the effect of PAK2 KD (El-Mansi *et al.*, 2023).

**FIGURE 6:**
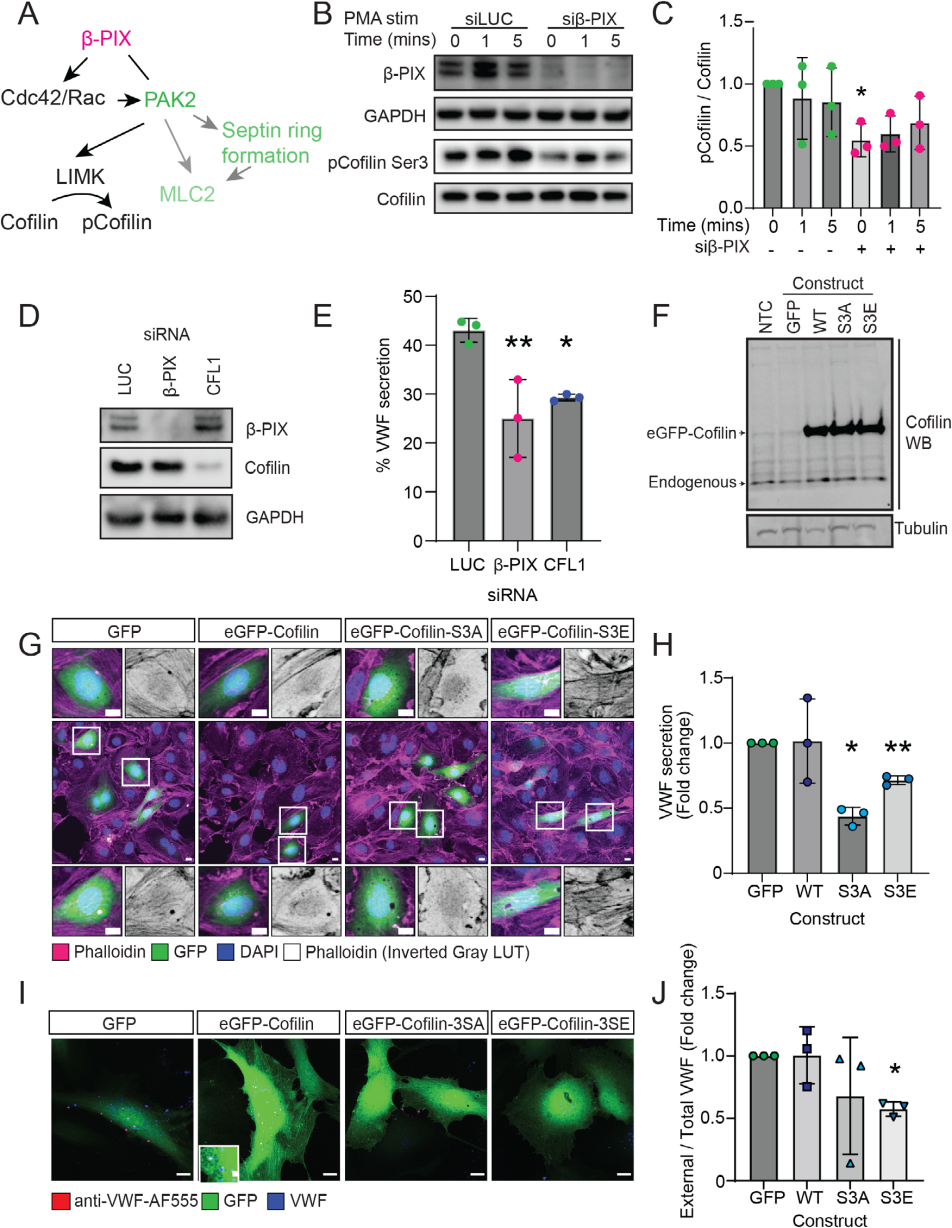
Cofilin is regulated by β-PIX, and its activity is required for VWF secretion. (A) Schematic of proteins regulated by β-PIX. (B and C) β-PIX was depleted in HUVEC using siRNA before stimulation with PMA for 0, 1, and 5 min. The levels of total and phospho-cofilin (Ser 3) were determined by (B) Western blot and (C) quantified *N* = 3. (D) HUVEC were transfected with control, Luciferase (LUC), β-PIX, or cofilin siRNA, and the efficiency of KD was determined by western blot. (E) KD HUVEC were exposed to PMA (100 ng/ml) for 30 min before the amount of VWF secreted into the culture media was quantified using a near-infrared fluorescent dot blot. *N* = 3. (F) HUVEC were transfected with either eGFP, WT eGFP-cofilin, eGFP-Cofilin-S3A, or eGFP- cofilin-S3E, and the protein levels were quantified by Western blot. (G) The effect of mutant cofilin expression on actin structure was determined by confocal microscopy, and (H), following the addition of PMA (100 ng/ml) for 30 min, the amount of VWF secreted into the culture media was quantified using Near-infrared fluorescent dot blot. *N* = 3. (I) HUVEC overexpressing WT and mutant cofilin were stimulated for 10 min in the presence of an anti-VWF-A555 conjugated antibody. Cells were then fixed and labeled for total VWF before confocal imaging. (J) The number of VWF exit sites was quantified in comparison to total VWF levels. *N* = 3. Data are presented as a fold change. Ratio paired *t* test. *p < 0.05 **p < 0.01.

Other potential mechanisms for controlling actin turnover are via regulation of Lim kinase and the actin severing protein cofilin (Edwards *et al.*, 1999; Figure 6A) From our proximity proteomics dataset we previously noted cofilin was within 20 nm of WPB during VWF release and from a high throughput siRNA screen, cofilin depletion in HUVEC reduces VWF release following stimulation with PMA or histamine epinephrine and IBMX (El-Mansi *et al.*, 2023). Cofilin's actin-severing function is inactivated by phosphorylation on Serine 3. We monitored cofilin phosphorylation on Ser 3 and noted a significant difference following β-PIX KD, suggesting cofilin regulation is important for controlling VWF secretion (Figure 6, B and C; Supplemental Figure S4). Using siRNA depletion, we verified the effect of cofilin on VWF secretion (Figure 6, D and E).

To further explore the role of cofilin, we overexpressed plasmids expressing full-length GFP-tagged cofilin (WT), constitutively active non-phosphorylatable cofilin (cofilin-GFP-S3A), or a dominant negative pseudophosphorylated version (cofilin-GFP-S3E; Figure 6F). Following expression of the S3A mutant, we noted changes in membrane ruffling at the leading edge, whilst the S3E mutant most closely resembled the actin phenotype noted on β-PIX KD (Figure 6G). Both mutants inhibited VWF release monitored by secretion assay (Figure 6H). Image analysis of PMA stimulated cofilin-GFP construct overexpressing cells determined that the S3E mutant reduced the number of WPB fusion sites detected by external VWF antibody labelling (Figure 6, I and J) Acute activation of cofilin by (30 min) treatment with a Lim kinase inhibitor (TH257) inhibited S3 phosphorylation (Supplemental Figure S5) and augmented PMA regulated VWF secretion at the lower doses, however this effect diminished at higher concentrations. Overall, this indicates that coordinated and timely actin severing is required for VWF release, most likely by remodeling the actin ring as it contracts, and that β-PIX regulates this process alongside septin ring recruitment.

## DISCUSSION

Using genetic and pharmacological approaches, we previously demonstrated that PAK2 signaling is essential for VWF secretion, requiring activation by both its kinase domain and Cdc42/Rac. Here, to identify additional therapeutic targets, we demonstrate that the PAK-interacting GEF, β-PIX, controls VWF exocytosis.

Our work indicates that β-PIX dependent actin remodeling potentially influences VWF exocytosis by several direct and indirect mechanisms which are not mutually exclusive. First, it directly influences the turnover and contractility of the secretory actomyosin ring; secondly by changing the composition of prominent cytoskeletal actin structures it might indirectly limit the availability of machinery necessary for actomyosin ring function; finally, by influencing the composition of cortical actin and improving accessibility to the plasma membrane it may alter the rate of organelle fusion.

Depletion of β-PIX using siRNA or overexpression of truncated and point mutants of β-PIX in HUVEC resulted in a distinct dominant negative actin phenotype, characterized by the presence of prominent F-actin stress fibers over the nucleus. This required the PAK-interacting SH3 domains and the Cdc42/Rac-interacting DH domain. The DH domain is necessary for GEF activity by binding Cdc42 and Rac, and a point mutation in this domain was sufficient to recapitulate the dominant negative feedback. This indicates that β-PIX acts to corral PAK2 via the SH3 domain, whereas the GEF domain provides a means to instigate this signaling cascade through activation of Cdc42/Rac.

There is good evidence for a role of GTPases Rac1/Cdc42 in WPB exocytosis. We noted a marked effect of pharmacological inhibition (IPA-3) of the Cdc42/Rac1 binding domain of PAK2 (El-Mansi *et al.*, 2023). Rac-1 depletion increases stress fiber formation in an analogous manner to the phenotype we noted in β-PIX KD and is required for epinephrine-induced WPB exocytosis (van Hooren *et al.*, 2014). Cdc42 is required for PAR-mediated exocytosis (Klarenbach *et al.*, 2003), and we also previously noted a relatively modest effect of Cdc42 depletion on PMA and Histamine/Epinephrine induced VWF secretion (El-Mansi *et al.*, 2023).

A variety of agents induce WPB mobilization and VWF secretion (Vischer *et al.*, 2000; Rondaij *et al.*, 2006). Agents that elevate intracellular cAMP initiate a more sustained release of VWF (Nightingale *et al.*, 2018), and this is accompanied by junctional strengthening and disassociation of stress fibers to maintain vascular barrier function (Vischer *et al.*, 2000). These include epinephrine, forskolin, or desmopressin—the latter, a modified form of vasopressin, is used therapeutically to manage von Willebrand's disease (Mannucci *et al.*, 1977). We and others have reported that phorbol esters and secretagogues, which utilize cAMP, require the nucleation and contraction of actomyosin coats/rings, which form on the WPB membrane post-fusion and promote cargo release in a force-driven process (Nightingale *et al.*, 2011; Nightingale *et al.*, 2018; McCormack *et al.*, 2020). While endothelial actomyosin rings are formed most effectively in response to cAMP, they are also observed (albeit less frequently and at later time points) during Ca^2+^ flux and injury (scratch wounding) responses, where their occurrence correlates with rigidity of the cell cortex (Mietkowska *et al.*, 2019).

Biochemical assays and live cell imaging demonstrated that depletion of β-PIX reduced the proportion of WPBs that fused with the PM and increased the time taken for GFP-VWF to be expelled. Other evidence for ineffective exocytosis was seen in the prolonged assembly and contraction kinetics of exocytic actomyosin rings/coats, which form post-fusion to aid the expulsion of ultralarge VWF multimers (Nightingale *et al.*, 2011). The kinetics of the exocytic defect are very similar to perturbation by actomyosin and septin inhibitors (Nightingale *et al.*, 2011; El-Mansi *et al.*, 2023; El-Mansi *et al.*, 2024). This therefore indicates both pre-fusion and post-fusion phenotypes.

Using KD and rescue experiments in conjunction with VWF secretion assays, we found that only the full-length Em-β-PIX was able to restore the defect caused by the depletion of endogenous β-PIX. Surprisingly, despite retaining the ability to bind PAK and Cdc42/Rac, constructs lacking the GID and CTR domains failed to rescue the phenotype. We propose that this failure could be explained by the altered spatial profile of β-PIX and subsequently its bound effectors (PAK and Cdc42/Rac). It has been proposed that other focal adhesion-associated proteins, such as Zyxin, are involved in remodeling of cortical actin (Han *et al.*, 2017); as such, the largely nuclear/perinuclear localization of Em-β-PIX (65-400) and Em-β-PIX (1-400) may preclude important actin remodeling events in the periphery. This is pertinent as WPB in the cell periphery are more mature, contain higher MW multimers (Nightingale *et al.*, 2009), and longer WPB are more likely to utilize an actin ring (McCormack *et al.*, 2020). Ultimately, these data suggest that multiple β-PIX interactions are required for optimal VWF secretion.

The actin structures necessary for secretion are dynamic, with actin rings lasting on average 20 s (Nightingale *et al.*, 2011). At this time, the ring forms (either by nucleation or by remodeling of existing actin filaments), contracts, and disassembles (Nightingale *et al.*, 2012). Our data demonstrates a crucial role for actin severing in VWF secretion and specifically for cofilin in this process downstream of β-PIX. Cofilin function is tightly regulated by phosphorylation (Bravo-Cordero *et al.*, 2013), and our data reveal that both constitutively active and dominant negative versions of cofilin inhibit VWF secretion. Acute activation of cofilin function via LIMK inhibition displayed a similarly mixed phenotype. This indicates that tight and timely regulation of cofilin is required for VWF secretion, with cofilin either remodeling existing actin to form the ring and/or remodeling actin during contraction and disassembly. Cofilin-mediated actin depolymerization has been shown to generate force (Sun *et al.*, 2010; Mseka and Cramer, 2011; Miklavc *et al.*, 2015), so this may represent an active role for cofilin in VWF release. In agreement with this, lamellar bodies from alveolar type II cells similarly recruit an exocytic actin coat, and expression of both cofilin S3A or S3E constructs inhibits release (Miklavc *et al.*, 2015). There is also the potential for broader effects of cofilin in changing the rigidity of cortical actin and affecting the rate of WPB fusion. Inhibition of actin polymerization using cytochalasin E causes an increase in the number of WPB fusion events (Nightingale *et al.*, 2011). It is therefore conceivable that regulation of global actin polymerization and exocytic actin ring function caused by changes in small GTPases and PAK2 function occur in conjunction.

Our work shows that β-PIX is required for septin ring regulation. This is likely due to a direct effect of PAK2 on septin function, although previous reports have also placed Cdc42 and its effector proteins (Borgs) as key mediators of septin function (Joberty *et al.*, 2001; Farrugia and Calvo, 2017). There are also potential roles for β -PIX in the regulation of lipid moieties essential for recruiting effectors to the relevant membranes. In neuroendocrine cells (Momboisse *et al.*, 2009), Rac-stimulated β-PIX promotes Phospholipase D (PLD) activity to generate fusogenic lipids essential for exocytosis. Moreover, both Rac1 and Cdc42 have been shown to activate Type I Phosphatidylinositol 4-Phosphate 5-Kinase (Weernink *et al.*, 2004), which synthesizes phosphatidylinositol 4,5-bisphosphate (PIP2). We and others have reported the importance of PIP2 in mediating WPB fusion (Nguyen *et al.*, 2020) and the recruitment of Myo1C (El-Mansi *et al.*, 2024), which links the actin ring to the PM. A role for septin recruitment via PIP2 is also well established (Zhang *et al.*, 1999; Babich *et al.*, 2008). Therefore, we consider it likely that β-PIX has pleiotropic roles in mediating exocytosis, which likely rely on coordinated control of septin and actin rings. We cannot rule out indirect effects on septin oligomerization, β-PIX-mediated remodeling of FAs, and F-actin stress fibers, which may be important for the dissolution of septins from actin filaments for use in exocytic processes.

Nearly 30 years ago, Manser *et al.*, described the essential role of β-PIX as a dual-specific GEF for Cdc42 and Rac (Manser *et al.*, 1998). Since then, much progress has been made to reveal its diverse functions. Our findings place β-PIX as an essential GEF supporting actomyosin-mediated expulsion of endothelial secretory granules. Future work should address the effect of pharmacological targeting β-PIX in vivo and determine translational potential to prevent thrombotic disease and, more broadly, other secretory pathologies

## MATERIALS AND METHODS

Request a protocol through *Bio-protocol*

### Cell culture

Human Umbilical Vein Endothelial Cells (HUVEC; PromoCell, Catalogue: 12203) from pooled donors were cultured and used as described previously (Michaux *et al.*, 2006).

### Immunofluorescence and western blotting

This was performed as described elsewhere (Nightingale *et al.*, 2011), and the commercial suppliers of antibodies used here are provided in Supplemental Table S1. For extracellular in situ labelling of Weibel-Palade body fusion sites (exit sites), (Stevenson *et al.*, 2017) HUVEC were stimulated for 10 min with culture media containing PMA (100 ng/ml) and anti-VWF-AF555 (Dako: 1 in 500). HUVEC were then washed in PBS twice before fixation in 4% PFA. Antibodies were conjugated using commercially available kits (Thermo Fischer Scientific Catalogue: A88065).

### Fixed and live cell imaging

Depending on the experimental aim, microscopy studies were performed using either the Zeiss LSM800 or the Nikon CSU-W1 SoRa spinning disk microscope with Z stack intervals ranging from 0.1–0.5 µm depending. Where necessary, image brightness and contrast were adjusted for clarity. In total, 3µg of plasmid DNA was transfected per 0.5 × 10^6^ cells. A table detailing the plasmids used for transient transfection is provided in the supplementary materials (Supplemental Table S2). Temporal color-coded maximum intensity projections were created using ImageJ and the Temporal Color Code tool in Hyperstacks.

### siRNA depletion of target proteins in HUVEC

ON-TARGETplus siRNA targeting ARHGEF7 (Catalogue: L-009616), Cofilin (Catalogue: L-012707), and siRNA targeted firefly luciferase transcripts were generated by Eurofins Genomics (sequence 5′ cgu-acg-cgg-aau-acu-ucg 3′). Transfection of HUVEC was performed by electroporation using the “HUVEC-old” program on the Amaxa Biosystems Nucleofector. Two rounds of 300 pM siRNA were needed to consistently achieve effective knockdown (KD) efficiencies ranging above 90% target protein depletion. Transfections would be performed on days 1 and 3 before assaying on day 5. For knockdown and rescue experiments, 3µg of plasmid DNA was transfected per 0.5 × 10^6^ cells 24 h after the second siRNA transfection.

### VWF secretion assays

VWF secretion assay and near-infrared (NIR)-fluorescent dot blot were performed as described in our previous research (El-Mansi *et al.*, 2023). To promote VWF secretion, we utilized VEGF (40 ng/ml), PMA (100 ng/ml), Epinephrine (100 µM), and Forskolin (10 µM). Forskolin and Epinephrine were supplemented with 100 µM IBMX (3-isobutyl-1-methylxanthine).

### Quantification of VWF exit sites

Using ImageJ (FIJI), confocal images were converted to an 8-bit Z stack before the background was subtracted with a 50 micron diameter rolling ball. The threshold of both total and extracellular VWF channels was adjusted automatically using the Bernsen Method. The total area of each channel was measured and presented as an external VWF as a fraction of total VWF. Data was then normalized to the GFP control.

### Statistical analysis

Statistical analysis was performed using GraphPad Prism software. When comparing two groups, an unpaired students *t* test was performed. When comparing groups that have been normalized (fold change), a ratio paired *t* test was performed. All error bars shown represent the standard error of the mean.

## Supporting information





Supporting Video 1 Movie S1 HUVEC were mock transfected with luciferase siRNA over two consecutive rounds. On the second round a Lifeact-GFP construct was also included. Cells were then stimulated with 100ng/ml PMA before imaging by spinning disk confocal microscopy; Scale bar 10µm.

Supporting Video 2 Movie S2 HUVEC were transfected with β-Pix siRNA over two consecutive rounds. On the second round a Lifeact-GFP construct was also included. Cells were then stimulated with 100ng/ml PMA before imaging by spinning disk confocal microscopy; Scale bar 10µm.

## Abbreviations used:

Cdc42: cell division cycle 42
DH: Dbl-homology domain
GEF: guanine nucleotide exchange factor
GID: GIT1/2 interaction domain
IBMX: 3-Isobutyl-1-methylxanthine
KD: knockdown
PAK2: P21 activated kinase 2
PIP2: phosphatidylinositol 4,5-bisphosphate
PKC: protein kinase C
PM: plasma membrane
SH3: Src homology 3
VEGF: vascular endothelial growth factor
VWF: von Willebrand factor
WPBs: Weibel-Palade bodies
β-PIX: Beta PAK-interacting exchange factor.
