# Supplementary material for: Endothelial β-PIX (ARHGEF7) supports actomyosin mediated expulsion of VWF through dynamic reorganization of the cytoskeleton
Source: Mol Biol Cell. 2026 May 4;37(6):ar49. doi: 10.1091/mbc.E25-10-0483 (PMC13200706; doi:10.1091/mbc.E25-10-0483)
Supplement: Supplementary file 1 [file mbc-37-ar49-s001.pdf]

Supplemental Materials

Molecular Biology of the Cell

El-Mansi *et al*.

Supplementary Materials for

Endothelial β-PIX (ARHGEF7) supports actomyosin mediated expulsion of VWF through dynamic reorganization of the cytoskeleton.

*Corresponding author. Email: s.elmansi@qmul.ac.uk and t.nightingale@qmul.ac.uk

This PDF file includes:

Table S1 and S2
Figs. S1, S2, S3, S4 and S5

Target name	Host species	Cat number	Supplier	Dilution	Method
**VWF**	Rabbit	A0082	Dako	1 in 10000	IF
**VWF**	Sheep	AHP062	Bio-Rad	1 in 10000	IF
**ARHGEF7/B-PIX**	Mouse	sc-393184	Santa Cruz	1 in 1000	WB
**TUBULIN**	Mouse	T5201	Sigma Aldrich	1 in 3000	WB
**GAPDH**	Mouse	60004-1-Ig	Protein Tech	1 in 3000	WB
**GAPDH**	Rabbit	10494-1-AP	Protein Tech	1 in 3000	WB
**anti Rabbit IgG NIR 800**	Donkey	A21057	Licor	1 in 15000	WB
**Anti-Mouse IgG NIR 680**	Goat	92668071	Licor	1 in 15000	WB
**GFP**	Rat	3H9	Chromotek	1 in 1000	WB
**Cofilin (Ser 3)**	Rabbit	3311	Cell Signaling	1 in 1000	WB
**Cofilin**	Rabbit	5175	Cell Signaling	1 in 1000	WB

Table S1: Details of antibodies used in the investigation.

Construct	Source	Citation
**LifeAct-GFP**	A kind gift from the Baum Laboratory.	Riedl J, Crevenna AH, Kessenbrock K, et al. Lifeact: a versatile marker to visualize F-actin. Nat Methods. 2008;5(7):605-607.
**GFP-VWF**	A gift from J. Voorberg and J.A. Van Mourik (Sanquin Research Laboratory, Amsterdam, The Netherlands).	Romani de Wit T, Rondaij MG, Hordijk PL, Voorberg J, van Mourik JA. Real-time imaging of the dynamics and secretory behavior of Weibel-Palade bodies. Arterioscler Thromb Vasc Biol. 2003;23(5):755-761.
**P.sel.lum.mCherry**	Our laboratory	Nightingale TD, White IJ, Doyle EL, et al. Actomyosin II contractility expels von Willebrand factor from Weibel-Palade bodies during exocytosis. J Cell Biol. 2011;194(4):613-629
**ARHGEF7-GFP constructs**	A kind gift from Anthony Davidson and Vassilis Koronakis	Davidson A, Tyler J, Hume P, Singh V, Koronakis V. A kinase-independent function of PAK is crucial for pathogen-mediated actin remodelling. PLoS Pathog. 2021 Aug 30;17(8):e1009902. doi: 10.1371/journal.ppat.1009902. PMID: 34460869; PMCID: PMC8432889.
**GFP-N1-Cofilin constructs**	pEGFP-N1 human cofilin WT and mutants were a gift from James Bamburg (Addgene plasmid # 50859, #50861 and #50860)	Garvalov BK, Flynn KC, Neukirchen D, Meyn L, Teusch N, Wu X, Brakebusch C, Bamburg JR, Bradke F. Cdc42 regulates cofilin during the establishment of neuronal polarity. J Neurosci. 2007 Nov 28;27(48):13117-29. doi: 10.1523/JNEUROSCI.3322-07.2007. PMID: 18045906; PMCID: PMC6673401.

Table S2: Source of plasmid DNA constructs used for the transfection of HUVEC.

.

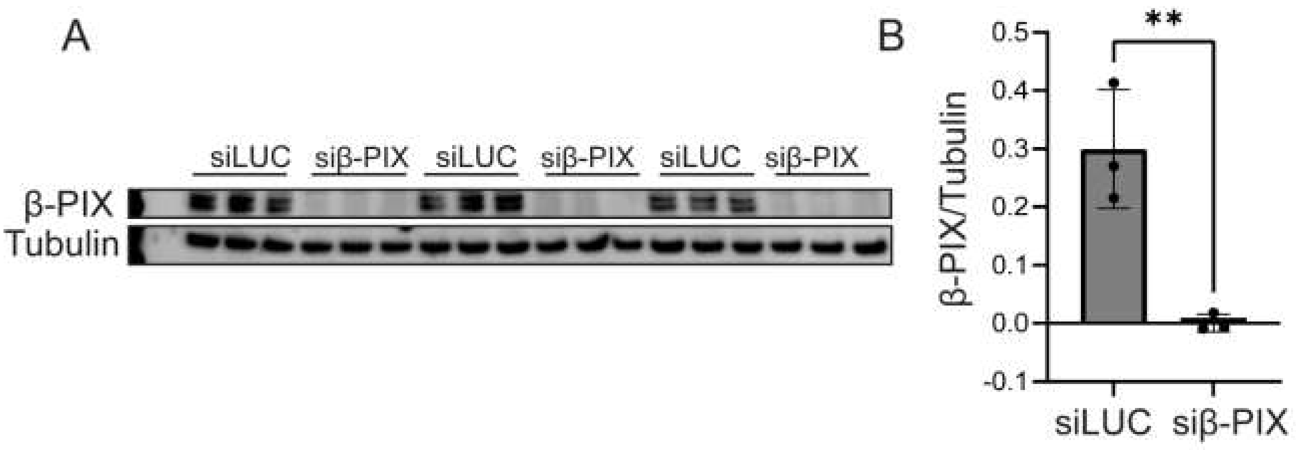

Figure S1: siRNA mediated depletion of β-PIX. (A) Immunoblotting of β-PIX and Tubulin in HUVEC transfected with 300pM siRNA. siLUC = siLuciferase control. N=3. (B) Densitometry confirmed effective KD. **P<0.01 Student t test.

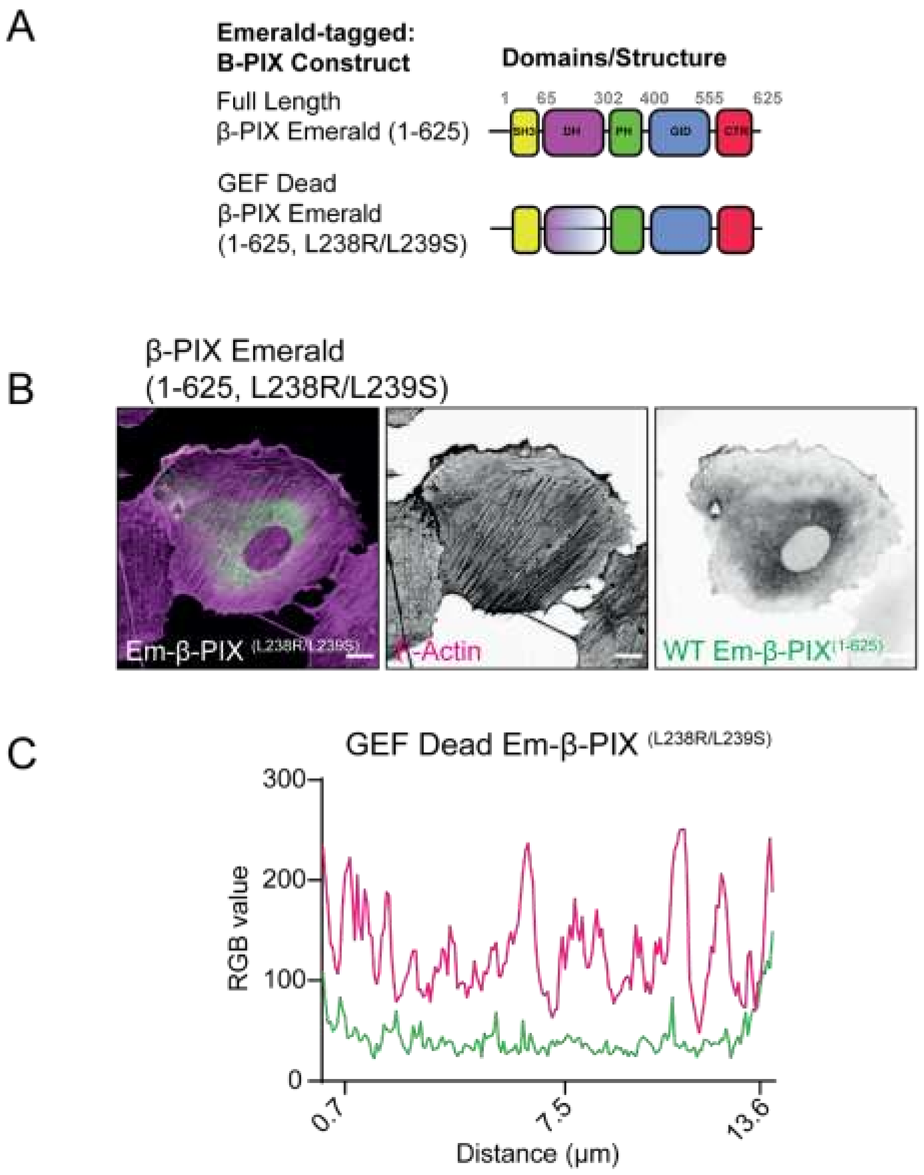

Figure S2. **(A)** Schematic representation of the domains and structure of Em-β-PIX$^{(1\text{-}165)}$ and Em-β-PIX$^{(L238R/L293S)}$ (GEF dead). **(B)** Confocal imaging and analysis of F-actin architecture determined that Em-β-PIX$^{(L238R/L293S)}$ expression phenocopied the effect of SH3 and SH3/DH truncated mutants **(C)** RGB profile plot of Em-β-PIX (Green) and F-Actin (Magenta) above the nucleus. Scale bars are 10 μm.

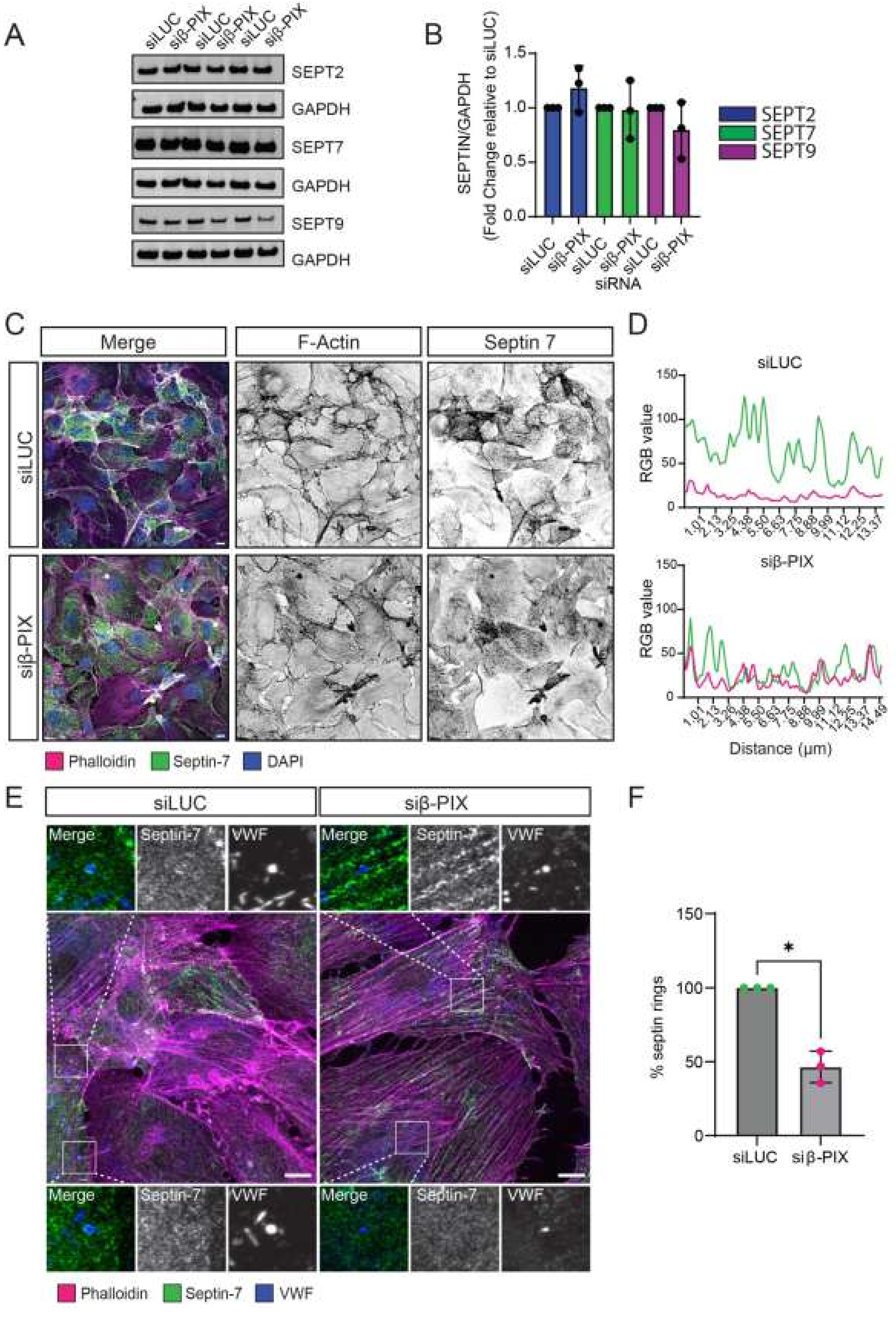

A
siLUC
siβ-PIX
siLUC
siβ-PIX
siLUC
siβ-PIX
SEPT2
GAPDH
SEPT7
GAPDH
SEPT9
GAPDH
B
SEPTIN/GAPDH
(Fold Change relative to siLUC)
siRNA
SEPT2
SEPT7
SEPT9
C
Merge
F-Actin
Septin 7
siLUC
siβ-PIX
Phalloidin
Septin-7
DAPI
D
siLUC
siβ-PIX
RGB value
Distance (µm)
E
siLUC
siβ-PIX
Merge
Septin-7
VWF
Phalloidin
Septin-7
VWF
F
% septin rings
siLUC
siβ-PIX
*

Figure S3. **(A)** siLUC or siβ-PIX treated HUVEC were western blotted to determine the levels of septin 2, 7 and 9 by western blot **(B)** Quantification of septin levels show no change in septin expression N=3. **(C)** siLUC or siβ-PIX treated HUVEC were fixed and labelled for confocal analysis (Phalloidin-Magenta, Septin-Green and DAPI-Blue) **(D)** there were subtle changes in overall septin localisation with a suggestion of altered stress fibre localisation. **(E)** siLUC or siβ-PIX treated HUVEC were stimulated with PMA (100ng/ml) for 7 min before fixing and labelling for confocal analysis (Phalloidin magenta, Septin-green and VWF-blue). **(F)** The number of septin rings were quantified and a significant reduction was noted following β-PIX depletion. N=3 Data is presented as a fold change. Ratio paired t test. *p<0.05. Scale bars are 10 μm.

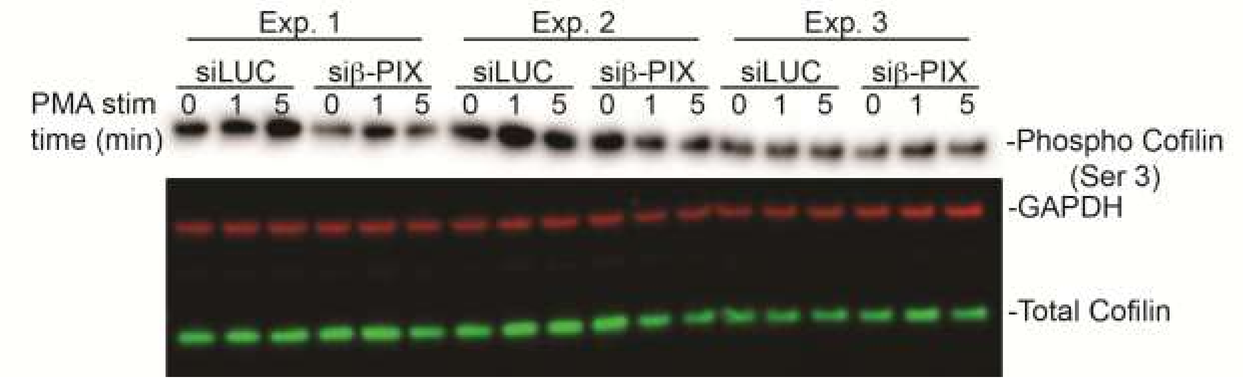

Figure S4. β-PIX was depleted in HUVEC using siRNA before stimulation with PMA (100 ng/ml) for 0, 1 and 5 min. The levels of total, phospho-cofilin (Ser 3) and GAPDH were determined by western blot; n=3.

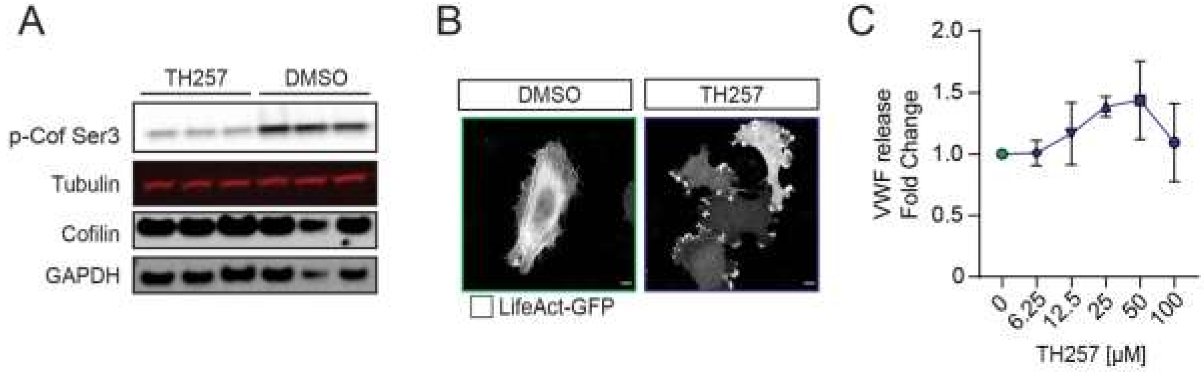

Figure S5. **(A)** HUVEC were treated with the LIM kinase inhibitor (50 μM) for 30 min at room temperature before being lysed and western blotted. TH257 inhibits phosphorylation at serine 3 leading to an activated state. **(B)** LifeAct-GFP expressing HUVEC were treated with the LIM kinase inhibitor (50 μM) for 30 min before fixation and imaging by confocal microscopy. TH257 causes a marked change in stress fibre formation. Scale bars are 10 μm. **(C)** HUVEC were treated with increasing concentration of the LIM kinase inhibitor for 30 min before stimulation with a combination of Histamine (100 μM), Adrenalin (10 μM) and IBMX (100 μM), VWF secretion was monitored by NIR dot blot.
