# Supplementary material for: Endothelial β-PIX (ARHGEF7) supports actomyosin mediated expulsion of VWF through dynamic reorganization of the cytoskeleton
Source: Mol Biol Cell. 2026 May 4;37(6):ar49. doi: 10.1091/mbc.E25-10-0483 (PMC13200706; doi:10.1091/mbc.E25-10-0483)
Supplement: Supplementary file 4 [file reviewer_reportE25-10-0483.pdf]

Endothelial β-PIX (ARHGEF7) supports actomyosin mediated expulsion of VWF through dynamic reorganization of the cytoskeleton.

Sammy El-Mansi, Karishma Deegan, and Thomas Nightingale

Corresponding author(s): Thomas Nightingale, Queen Mary University of London

Review Timeline:

Submission Date:	2025-10-10
Editorial Decision:	2025-11-21
Revision Received:	2026-03-02
Editorial Decision:	2026-03-31
Revision Received:	2026-04-09
Accepted:	2026-04-11

Editor-in-Chief: John Aitchison

Transaction Report:

(Note: With the exception of the correction of typographical or spelling errors that could be a source of ambiguity, letters and reports are not edited. The original formatting of letters and referee reports may not be reflected in this compilation.)

RE: Manuscript #E25-10-0483
TITLE: Endothelial β-PIX (ARHGEF7) supports actomyosin mediated expulsion of VWF through dynamic reorganization of the cytoskeleton.

Dear Dr. Nightingale:

The review of your manuscript is now complete. As you will see the three reviewers thought that the study was of interest but raised a number of significant issues which would need to be addressed in a revised manuscript. I would then send the revised manuscript to the original reviewers to ensure that their concerns had been satisfactorily addressed.

I look forward to seeing a revised manuscript in due course.

Sincerely,

Robert Parton
Monitoring Editor
Molecular Biology of the Cell

Dear Dr. Nightingale,

The review of your manuscript, referenced above, is now complete. The Monitoring Editor has decided that your manuscript is not acceptable for publication at this time, but may be deemed acceptable after specific revisions are made, as described in the Monitoring Editor's decision letter above and the reviewer comments below.

A reminder: Please do not contact the Monitoring Editor directly regarding your manuscript. If you have any questions regarding the review process or the decision, please contact the MBoC Editorial Office (mboc@ascb.org).

When submitting your revision include a rebuttal letter that details, point-by-point, how the Monitoring Editor's and reviewers' comments have been addressed. (The file type for this letter must be "rebuttal letter"; do not include your response to the Monitoring Editor and reviewers in a "cover letter.") Please bear in mind that your rebuttal letter will be published with your paper if it is accepted, unless you have opted out of publishing the review history.

Authors are allowed 90 days to submit a revision. If this time period is inadequate, please contact us at mboc@ascb.org.

Revised manuscripts are assigned to the original Monitoring Editor whenever possible. However, special circumstances may preclude this. Also, revised manuscripts are often sent out for re-review, usually to the original reviewers when possible. The Monitoring Editor may solicit additional reviews if it is deemed necessary to render a completely informed decision.

In preparing your revised manuscript, please follow the instruction in the Information for Authors (www.molbiolcell.org/info-for-authors). In particular, to prepare for the possible acceptance of your revised manuscript, submit final, publication-quality figures with your revision as described.

To submit the rebuttal letter, revised manuscript, and figures, use this link: Link Not Available

MBoC offers the option to publish your work with immediate open access. Open access can increase the discoverability and usability of your research. If you would like to publish your paper with immediate open access but did not select this option during initial submission, please contact the editorial office at mbc@ascb.org.

Authors of Articles and Brief Communications are also encouraged to create a short video abstract to accompany their article when it is published. These video abstracts, known as Science Sketches, are up to 2 minutes long and will be published on YouTube and then embedded in the article abstract. Science Sketch Editors on the MBoC Editorial Board will guide you as you prepare your video. Information about how to prepare and submit a video abstract is available at www.molbiolcell.org/science-sketches. Please contact mboc@ascb.org if you are interested in creating a Science Sketch.

Sign up for MBoC's new content alerts here https://tinyurl.com/3bcsyk3e

Thank you for submitting your manuscript to MBoC. We look forward to receiving your revised paper.

Sincerely,

Eric Baker
Journal Production Manager
MBoC Editorial Office
mbc@ascb.org

Reviewer #1 (Remarks to the Author):

This study utilizes siRNA knockdown and mutant protein expression to analyze the role of beta-PIX, a GEF for the small GTPases Rac1 and Cdc42, in actin cytoskeleton remodeling and von-Willebrand factor (VWF) secretion in human umbilical vein endothelial cells (HUVEC). The authors show that beta-PIX knockdown increase the number of actin stress fibers (RhoA effect?), reduces the relative number of exocytotic fusion events of Weibel-Palade bodies (WPB), the VWF storage organelles, and prolongs the lag time between WPB fusion with the plasma membrane and VWF expulsion into the extracellular milieu. As shown earlier by the authors, the latter is mediated by contraction of an actomyosin ring that forms around fused WPB, thereby supporting the release of very large VWF multimers. The authors conclude that in the course of VWF secretion, beta-PIX functions in controlling, presumably through Cdc42/Rac activation, the activity of p21 activated kinase 2, which has previously been shown to be required for VWF expulsion.

The authors employ several approaches, such as biochemical assays to quantify VWF secretion, high resolution and live cell microscopy, to document the effects of beta-PIX manipulation on F-actin and WPB dynamics. The study is well performed and as such rather descriptive as no attempts to reveal the underlying mechanism of beta-PIX action have been made. It would be interesting to learn whether beta-PIX indeed acts through Cdc42 and/or Rac (are activity levels altered following KD, can active Cdc42/Rac restore the rapid VWF expulsion in beta-PIX KD cells) and whether the inhibitory effect of beta-PIX KD on the number of WPB fusions (Fig 3D, a pre-fusion phenotype) is due to a more rigid cortical cytoskeleton. The latter is more difficult to analyze but should at least be considered.
Other points:
1. Throughout the study, PMA is used as a stimulus to induce WPB exocytosis and VWF secretion. This is a rather non-physiological stimulus. The authors argue that the number of WPB fusion events with actomyosin rings is higher following stimulation with cAMP rising agonists as compared to those elevating intracellular Ca. This presumably precluded the use of Ca-rising agonists in this study. However, it would be helpful to also show the effect of beta-PIX knockdown on actomyosin ring formation and/or VWF expulsion following a more physiological cAMP agonist, such as adrenalin or desmopressin or even forskolin - at least in one of the assays employed by the authors.
2. Figure 1. The IF stainings are of poor quality. Why are hardly any WPB visible (anti-VWF staining), even in the control (siLUC) scenario? Is the number of WPB per cell affected by beta-PIX KD?
3. Figure 3D. Do only 12.5% of all WPB fuse within the 5 min time interval after PMA stimulation? This number appears very low. Is this the result of PMA used as stimulants?
4. Figure 3E. KD of beta-PIX increases the lag time between WPB fusion (P.sel.lum.mCherry release) and VWF expulsion. Is this increase similar to the one observed following actomyosin inhibition?
5. Figure 4E,F. Data shown here are not mentioned in the text.
6. Figure 5A. Why is siRNA mediated KD of beta-PIX shown again? Is there a difference to the experiment shown in Fig S1?
7. Figure 5D. VWF secretion is analyzed by a dot blot assay. Why not using the well-established and more accurate VWF ELISA?
8. Discussion. The authors speculate that septin ring formation could be controlled by beta-PIX. This could be tested and would shed some light on the mechanism.

Reviewer #2 (Remarks to the Author):

See attached.

Reviewer #3 (Remarks to the Author):

Title: Endothelial β-PIX (ARHGEF7) supports actomyosin mediated expulsion of VWF through dynamic reorganization of the cytoskeleton
Summary: This study by El-Mansi et al., identifies the guanine nucleotide exchange factor β-PIX (ARHGEF7) as a previously unrecognised regulator of endothelial Weibel-Palade body (WPB) exocytosis and von Willebrand factor (VWF) release. Using siRNA depletion, live-cell imaging, biochemical assays, and domain-specific β-PIX mutants, the authors show that β-PIX is required for both cytoskeletal remodelling and efficient actomyosin-dependent release of VWF. β-PIX knockdown disrupts dynamic actin architecture, reduces PMA-induced membrane ruffling, and promotes formation of aberrant stress fibres. Functionally, β-PIX depletion lowers the proportion of WPBs that fuse with the plasma membrane, significantly delays VWF expulsion, and prolongs the lifetime of exocytic actomyosin rings. While truncated β-PIX constructs indicate that both the PAK-

interacting SH3 domain and Cdc42/Rac-activating DH domain are necessary for cytoskeletal control, only full-length β-PIX can rescue VWF secretion-highlighting the importance of its C-terminal localisation domains. Overall, the data support a model in which β-PIX coordinates Cdc42/Rac activation and PAK2-dependent actin dynamics to facilitate both pre-fusion WPB positioning and post-fusion actomyosin ring contraction. The authors propose β-PIX as an alternative and potentially more tractable therapeutic target within the PAK2 cytoskeletal signalling pathway for regulating pathological VWF release.

Major Comments

- The authors should provide independent physiological means for stimulating the Human Umbilical Vein Endothelial Cells (HUVEC). Use of PMA is mostly artificial. Consider stressing the cells (metabolic stress) to increase DAG, or over-express DAG. This should complement the data in figure 1 and reveal whether decreased levels of β-PIX prevent cortactin remodelling in response to increase in DAG levels.
- The authors indicated that truncation of the GID and CTR resulted in nuclear localisation of Em-β-PIX in Figure 2. However, there is no nuclear staining to support the nuclear re-distribution of PIX.
- The use of electrophysiology or other independent fusion assays is needed to complement and support the P.selectin luminal domain mCherry data used by the authors in figure 3 to make strong claims on β-PIX expression impacting fusion. The authors should consider pHluorin assays or other release assays to evaluate fusion. Also, does the GFP tag affect the expulsion of VWF? If smaller tags are used, does this affect the release efficiency?
- The authors should consider providing further details on how β-PIX regulates VWF release such as how Cdc42/Rac expression levels impact on β-PIX function. This is to drive the manuscript to provide more precise mechanistic answers.

Minor Comments

- The authors should rephrase the result title for figure 4, to better reflect what the data shows.

Rebuttal letter

We thanks the reviewers for their constructive comments and we have responded to them as follows:

The role of cdc42/Rac

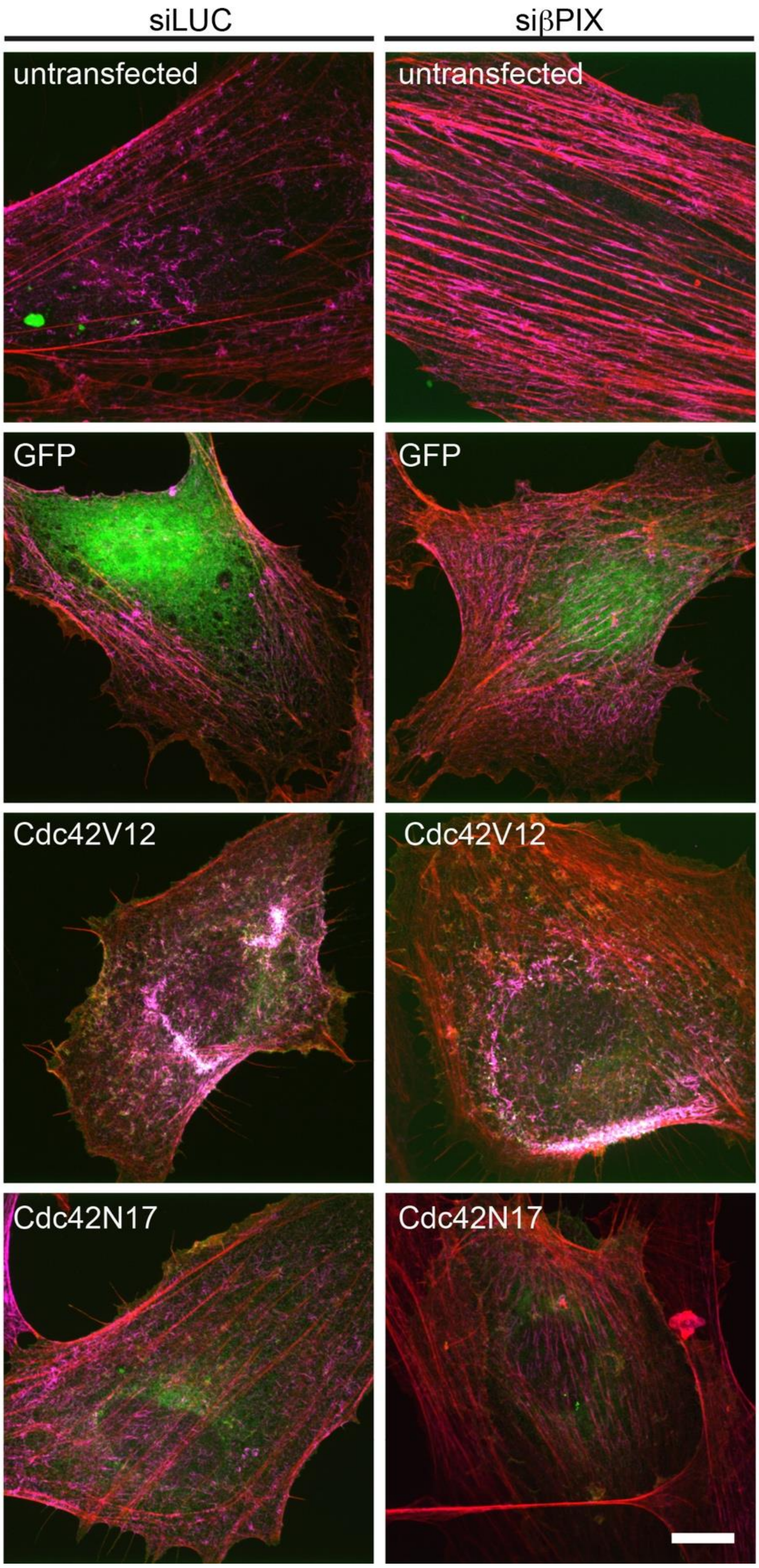

Rebuttal Figure 1 HUVEC were transfected over 2 rounds with siRNA targeting luciferase (siLUC) or (β-Pix). At the second round the cells were also either mock transfected or transfected with GFP, Cdc42 v12GFP or Cdc42 n17GFP. Cells were fixed and labelled for immunofluorescence (actin-red, cortactin magenta). There was notable changes in actin structure but transfection efficiency was insufficient to allow VWF secretion assays. Scale bar 10μm.

Reviewer #1 *It would be interesting to learn whether beta-PIX indeed acts through Cdc42 and/or Rac (are activity levels altered following KD, can active Cdc42/Rac restore the rapid VWF expulsion in beta-PIX KD cells)…* Reviewer # 3 *The authors should consider providing further details on how β-PIX regulates VWF release such as how Cdc42/Rac expression levels impact on β-PIX function. This is to drive the manuscript to provide more precise mechanistic answers.*

Previous work has shown that β-PIX regulates Rac in endothelial cells[1]; and in other cell types β-PIX has been shown to regulate cdc42[2]. Both Rac-1 and cdc42 have been shown to regulate WPB exocytosis to varying extents[3-5]. Monitoring Rac-1/cdc42 activity following β-PIX depletion therefore does not allow differentiation between the GTPase activity that is important for the actin stress fibre formation, or the actin polymerisation that forms the actomyosin ring. Perhaps, as suggested, the best way to specifically show a role in the actomyosin ring function is to rescue the WPB exocytosis defect. We attempted to rescue the fusion phenotype in KD cells using constitutively active (v12) or dominant negative (n17) CDC42 (Rebuttal Fig.1). Although expression of these constructs caused demonstrable differences in actin structures the constructs expressed poorly, precluding analysis by VWF secretion assay. Rac rescue experiments were similarly inefficient. Even with sufficient expression of the constructs a rescue approach would likely have been challenging as aberrant actin structures formed due to constitutively active CDC42 and RAC are likely to sequester key machinery.

We have not included the overexpression data in the paper as it is inconclusive, but we have included additional discussion of the role of CDC42 and Rac (lines 254-260) in WPB exocytosis.

Additional details of mechanism and importance of β-PIX

Reviewer #1 *The study is well performed and as such rather descriptive as no attempts to reveal the underlying mechanism of beta-PIX action have been made….* Reviewer#1 *Discussion. The authors speculate that septin ring formation could be controlled by beta-PIX. This could be tested and would shed some light on the mechanism…..*Reviewer #3 *The authors should consider providing further details on how β-PIX regulates VWF release*

To further delineate the pathway associated with β-PIX activity we have included additional data to include the downstream role of septin, LIM kinase (Fig. S3 & S4) and cofilin (Fig. 6) see (lines 203-233).

Alternate more physiological stimuli

Reviewer #1 *Throughout the study, PMA is used as a stimulus to induce WPB exocytosis and VWF secretion. This is a rather non-physiological stimulus. The authors argue that the number of WPB fusion events with actomyosin rings is higher following stimulation with cAMP rising agonists as compared to those elevating intracellular Ca. This presumably precluded the use of Ca-rising agonists in this study. However, it would be helpful to also show the effect of beta-PIX knockdown on actomyosin ring formation and/or VWF expulsion following a more physiological cAMP agonist, such as adrenalin or desmopressin or even forskolin - at least in one of the assays employed by the authors…..* Reviewer #3 *The authors should provide independent physiological means for stimulating the Human Umbilical Vein Endothelial Cells (HUVEC). Use of PMA is mostly artificial. Consider stressing*

the cells (metabolic stress) to increase DAG, or over-express DAG. This should complement the data in figure 1 and reveal whether decreased levels of β-PIX prevent cortactin remodelling in response to increase in DAG levels.

As suggested, we have incorporated additional secretagogues into our secretion assay (Fig. 5B-D). We noted the same trend with all secretagogues although only results with forskolin and VEGF stimulation reached significance (lines 188-192).

Difficulty in visualising WPB in Fig.1

Reviewer #1 *Figure 1. The IF stainings are of poor quality. Why are hardly any WPB visible (anti-VWF staining), even in the control (siLUC) scenario? Is the number of WPB per cell affected by beta-PIX KD?.....* Reviewer #2 *Figure 1: The immunofluorescence staining of vWF (blue) may be unnecessary, as it reduces image clarity. Consider removing the blue channel and clearly stating in the legend that the white (or merged) regions represent membrane ruffles.*

The difficulty in seeing the WPB reflects the low magnification of the images used and the blue LUT used for the VWF channel. We did not notice any difference in WPB number following β-PIX depletion. We have included a version of the image showing the VWF channel in grey alongside quantification of WPB number (Rebuttal Fig. 2). In Fig.1 we have removed the VWF channel as suggested, the primary focus of the first figure being the actin cytoskeleton. We have improved the labelling in the legend (line 402).

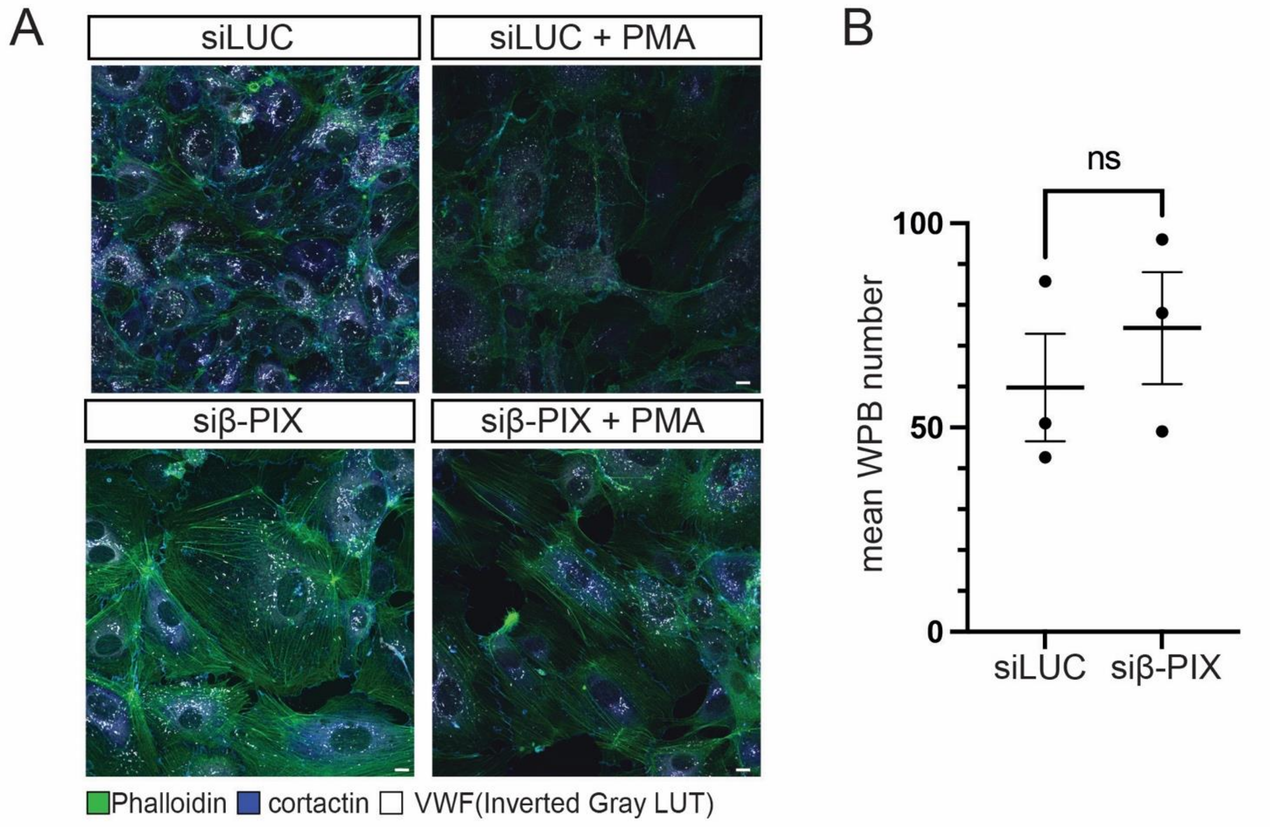

Rebuttal Figure 2 (A) Control (LUC) and β-PIX KD cells were left untreated or stimulated with PMA for 5 minutes before fixation, permeabilization and IF staining. Visualisation of F-Actin (green) cortactin (blue) and DAPI (grey) by confocal microscopy highlighted the presence of actin ruffles in control cells treated with PMA but not β-PIX KD cells. Moreover β-PIX KD cells displayed prominent dorsal F-Actin stress fibres under both conditions. **(B)**

The mean number of WPB in Control (LUC) and β-PIX KD cells was quantified over N=3 experiments. No significant difference in WPB was noted.

The role of cortical actin

Reviewer #1 *Whether the inhibitory effect of beta-PIX KD on the number of WPB fusions (Fig 3D, a pre-fusion phenotype) is due to a more rigid cortical cytoskeleton. The latter is more difficult to analyze but should at least be considered.*

Analyse of the effects on the rigidity of the cortical cytoskeleton are challenging to assess; we have included this as a potential mechanism for limiting WPB release in the discussion (lines 243-245).

The magnitude of WPB exocytosis with PMA

Reviewer #1 *Figure 3D. Do only 12.5% of all WPB fuse within the 5 min time interval after PMA stimulation? This number appears very low. Is this the result of PMA used as stimulants?*

PMA results in a more gradual release of WPB over a longer period of time whereas Histamine causes a more rapid release of a smaller subset of WPB; for example at 5 min there are 37% fewer exit sites in PMA stimulated cells compared to Histamine[6]. The fusion reported in Fig.3D is a % of all WPB present in the cell, this includes immature WPB, anchored in the cell but not competent to fuse and any punctae that are present in the secretory pathway including at the Golgi. 5 minutes is a convenient time point where a difference following KD can be noted robustly.

β-Pix kd vs effect of actin inhibitors

Reviewer #1 *Figure 3E. KD of beta-PIX increases the lag time between WPB fusion (P.sel.lum.mCherry release) and VWF expulsion. Is this increase similar to the one observed following actomyosin inhibition?*

This increase is similar to what we have previously noted[7]. As suggested, we have added this information in the text to clarify the phenotype (lines 276-277).

The validity of the dot blot assay vs VWF ELISA

Reviewer #1 *Figure 5D. VWF secretion is analyzed by a dot blot assay. Why not using the well-established and more accurate VWF ELISA?*

We previously developed and utilised the dot blot as a more convenient and efficient way of monitoring VWF secretion for screening assays and for monitoring VWF in mouse plasma samples. It uses much less secreted media/plasma (3 compared to 100µl). We have compared its accuracy and validity to the ELISA in previously published work[3]. We have included the figure here for reference (Rebuttal Fig.3) and have provided the reference in the text.

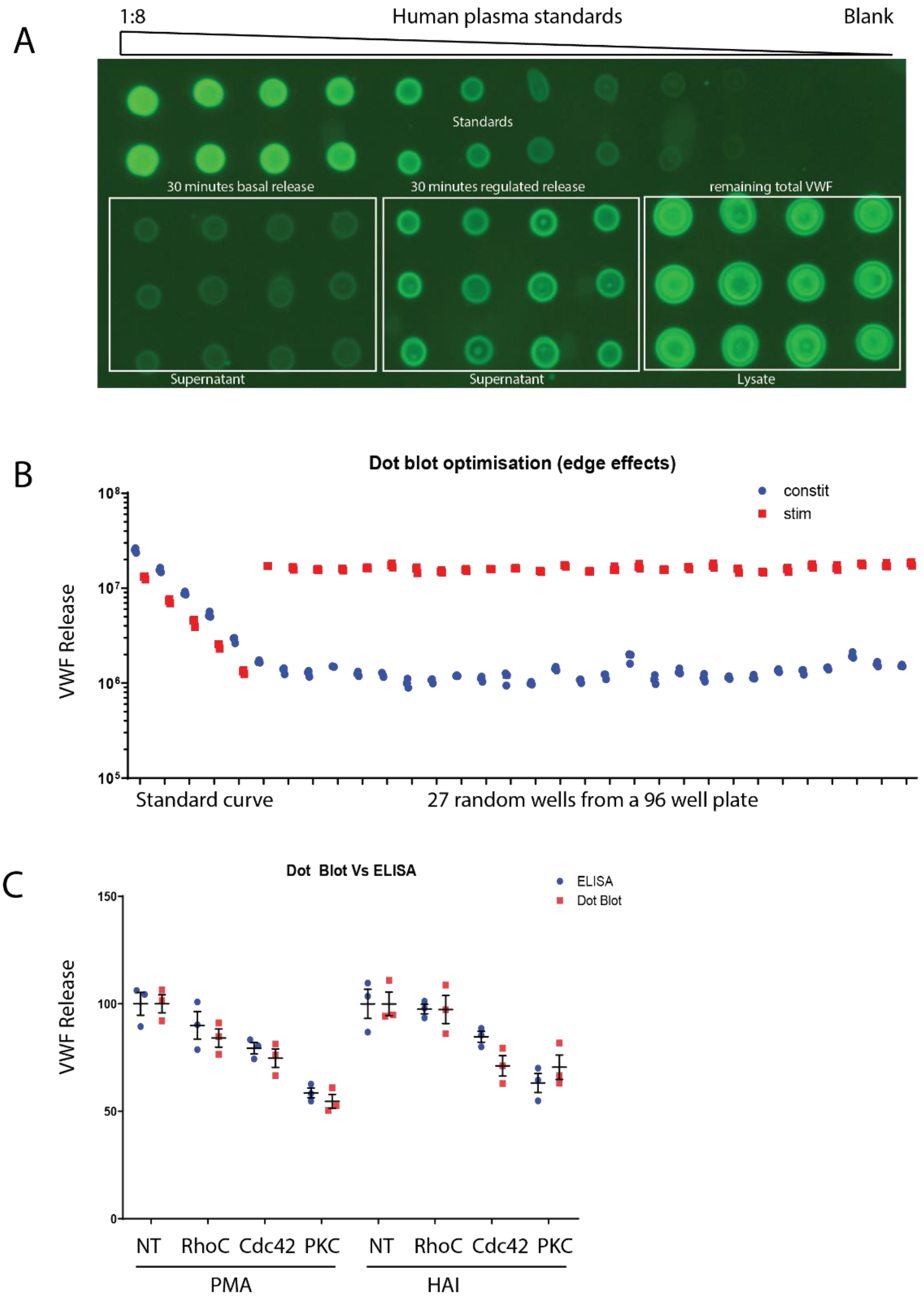

Rebuttal Figure 3. Optimization of the fluorescent dot blot. (A) Example blot. VWF was detected with an anti-VWF antibody, signal amplified using biotin conjugated antibody, and 800 IrDye labelled streptavidin. **(B)** No edge effects were determined **(C)** No significant differences were detected when the fluorescent dot blot was compared to a conventional VWF ELISA following KD of RhoC, Cdc42 and PKC**.**

Differential effects of β PIX rescue on actomyosin ring vs actin filament structure
Reviewer #2 *One point that could be further discussed concerns the interpretation of the mutant constructs. When endogenous β PIX is present (e.g., Figure 2 and LUC control Figure 5D), the ΔSH2 constructs appear to act as dominant negatives, preventing stress fibre depolymerization—a process the authors propose is necessary for actin ring dynamics. In contrast, the other two mutants are mislocalized and therefore do not interfere with endogenous β PIX function. Given this, if actin depolymerization is indeed required for vWF secretion, why does overexpression of the two ΔSH2 mutants in LUC control cells (where endogenous β PIX is present) not affect vWF secretion? Could these mutants also be used to directly investigate actin ring formation or depolymerization dynamics?*

We were similarly surprised that that the two ΔSH2 mutants did not rescue the exocytic actomyosin ring. We hypothesise this reflects the fact that additional domains are required to correctly localise the GEF for actomyosin ring function while these domains are not necessary for the role in stress fibre formation. We have included extra text to clarify this point (lines 199-201 and line 282-284).

The validity of the live cell imaging exocytosis assay

Reviewer #3 *The use of electrophysiology or other independent fusion assays is needed to complement and support the P.selectin luminal domain mCherry data used by the authors in figure 3 to make strong claims on β-PIX expression impacting fusion. The authors should consider pHluorin assays or other release assays to evaluate fusion. Also, does the GFP tag affect the expulsion of VWF? If smaller tags are used, does this affect the release efficiency?*

The P-selectin luminal domain mcherry approach was originally described in Nightingale et al 2011 and has been used by ours and other groups alongside VWF GFP for a number of years to monitor the timing of fusion and the effects of key regulatory proteins [3,6-11]. In the VWF GFP construct the GFP tag replaces the A2 domain of VWF[12], the A2 domain is 38kDa and the GFP tag is a little smaller, 28kDa, leading to little overall change in the size of the protein. VWF GFP has been shown to fold, multimerise and traffic as normal and is a good proxy for endogenous VWF.

We have previously used antibody in the surrounding media to detect VWF as it is released as “exit sites” in a time resolved manner, the broader kinetics of exit site formation mirror the dynamics as assessed by live cell imaging[6] supporting the validity of our live cell imaging approach. As suggested, we have corroborated our data detailing a role of cofilin in VWF release in Fig. 6 with an exit site assay.

Labelling errors
Reviewer #1 *Figure 4E,F. Data shown here are not mentioned in the text.*

Apologies E and F are now mentioned in the text.

Reviewer #1 *Figure 5A. Why is siRNA mediated KD of beta-PIX shown again? Is there a difference to the experiment shown in Fig S1?*

There is no difference in the experiment this was just to show the extent of KD for that particular experiment. We have removed it for clarity.

Reviewer #2 *Figures 3 and 4: Please indicate which images correspond to control versus siRNA-treated conditions.*

Additional labelling has been added to the figures as suggested

Reviewer #2 *Supplementary – Tables – Change format so that text fits more appropriately*

This has been changed

Reviewer #2 *Supplementary – Figure S2 – Change labels – ABC to appropriate size*
This has been changed

Reviewer #3 *The authors indicated that truncation of the GID and CTR resulted in nuclear localisation of Em-β-PIX in Figure 2. However, there is no nuclear staining to support the nuclear re-distribution of PIX.*

We have added a nuclear stain to the figure for clarity.

Reviewer #3 *The authors should rephrase the result title for figure 4, to better reflect what the data shows.*

As suggested, we have amended the title for figure 4 (see line 172)

References

1 Birukova, A. A., Malyukova, I., Mikaelyan, A., Fu, P. & Birukov, K. G. Tiam1 and betaPIX mediate Rac-dependent endothelial barrier protective response to oxidized phospholipids. *J Cell Physiol* **211**, 608-617, doi:10.1002/jcp.20966 (2007).

2 Manser, E. *et al.* PAK kinases are directly coupled to the PIX family of nucleotide exchange factors. *Mol Cell* **1**, 183-192, doi:10.1016/s1097-2765(00)80019-2 (1998).

3 El-Mansi, S. *et al.* Proximity proteomics identifies septins and PAK2 as decisive regulators of actomyosin-mediated expulsion of von Willebrand factor. *Blood* **141**, 930-944, doi:10.1182/blood.2022017419 (2023).

4 Klarenbach, S. W., Chipiuk, A., Nelson, R. C., Hollenberg, M. D. & Murray, A. G. Differential actions of PAR2 and PAR1 in stimulating human endothelial cell exocytosis and permeability: the role of Rho-GTPases. *Circ Res* **92**, 272-278, doi:10.1161/01.res.0000057386.15390.a3 (2003).

5 van Hooren, K. W. *et al.* Phosphatidylinositol-3,4,5-triphosphate-dependent Rac exchange factor 1 regulates epinephrine-induced exocytosis of Weibel-Palade bodies. *Journal of thrombosis and haemostasis : JTH* **12**, 273-281, doi:10.1111/jth.12460 (2014).

6 Nightingale, T. D. *et al.* Tuning the endothelial response: differential release of exocytic cargos from Weibel-Palade bodies. *Journal of thrombosis and haemostasis : JTH* **16**, 1873-1886, doi:10.1111/jth.14218 (2018).

7 Nightingale, T. D. *et al.* Actomyosin II contractility expels von Willebrand factor from Weibel-Palade bodies during exocytosis. *The Journal of cell biology* **194**, 613-629, doi:jcb.201011119 [pii]10.1083/jcb.201011119 (2011).

8 El-Mansi, S. *et al.* Myosin-1C augments secretion of von Willebrand factor by linking contractile actomyosin machinery to the plasma membrane. *Blood Adv*, doi:10.1182/bloodadvances.2024012590 (2024).

9 Han, X. *et al.* Zyxin regulates endothelial von Willebrand factor secretion by reorganizing actin filaments around exocytic granules. *Nature communications* **8**, 14639, doi:10.1038/ncomms14639 (2017).

10 Qiao, Z. *et al.* Clusterin maintains hemostatic equilibrium by stabilizing VWF multimers in plasma. *Blood*, doi:10.1182/blood.2025030643 (2026).

11 Stevenson, N. L. *et al.* Clathrin-mediated post-fusion membrane retrieval influences the exocytic mode of endothelial Weibel-Palade bodies. *Journal of cell science* **130**, 2591-2605, doi:10.1242/jcs.200840 (2017).

12 Romani de Wit, T., Rondaij, M. G., Hordijk, P. L., Voorberg, J. & van Mourik, J. A. Real-time imaging of the dynamics and secretory behavior of Weibel-Palade bodies.

Arteriosclerosis, thrombosis, and vascular biology **23**, 755-761, doi:10.1161/01.ATV.0000069847.72001.E8 (2003).

RE: Manuscript #E25-10-0483R
TITLE: "Endothelial β-PIX (ARHGEF7) supports actomyosin mediated expulsion of VWF through dynamic reorganization of the cytoskeleton."

Dear Dr. Nightingale,

The review of your revised manuscript is now complete. As you will see there were some minor comments raised by Reviewer 1 that should be addressed. Once these revisions are submitted and verified, we will proceed with formal acceptance.

Thank you again for your contribution to MBoC.

Sincerely,

Robert Parton
Monitoring Editor
Molecular Biology of the Cell

Dear Dr. Nightingale,

The review of your manuscript, referenced above, is now complete. The Monitoring Editor has decided that your manuscript requires minor revisions before it can be published in Molecular Biology of the Cell, as described in the Monitoring Editor's decision letter above and the reviewer comments (if any) below.

A reminder: Please do not contact the Monitoring Editor directly regarding your manuscript. If you have any questions regarding the review process or the decision, please contact the MBoC Editorial Office (mboc@ascb.org).

When submitting your revision include an authors' response letter that details, point-by-point, how the Monitoring Editor's and reviewers' comments have been addressed. (The file type for this letter must be "response letter"; do not include your response to the Monitoring Editor and reviewers in a "cover letter.") Please bear in mind that your response letter will be published with your paper if it is accepted, unless you have opted out of publishing the review history.

Authors are allowed 15 days to submit a revision. If this time period is inadequate, please contact us immediately at mboc@ascb.org.

In preparing your revised manuscript, please follow the instructions in the Information for Authors (www.molbiolcell.org/info-for-authors). In particular, to prepare for the possible acceptance of your revised manuscript, submit final, publication-quality figures with your revision as described.

To submit the response letter, revised version, and figures, please use this link (please enable cookies, or cut and paste URL):
Link Not Available

MBoC offers the option to publish your work with immediate open access. Open access can increase the discoverability and usability of your research. If you would like to publish your paper with immediate open access but did not select this option during initial submission, please contact us at mbc@ascb.org.

Authors of Articles and Brief Communications whose manuscripts have returned for minor revision ("provisional acceptance") are encouraged to create a short video abstract to accompany their article when it is published. These video abstracts, known as Science Sketches, are up to 2 minutes long and will be published on YouTube and then embedded in the article abstract. Science Sketch Editors on the MBoC Editorial Board will guide you as you prepare your video. Information about how to prepare and submit a video abstract is available at www.molbiolcell.org/science-sketches. Please contact mboc@ascb.org if you are interested in creating a Science Sketch.

Thank you for submitting your manuscript to MBoC. Please do not hesitate to contact this office if you have any questions.

Sign up for MBoC's new content alerts here https://tinyurl.com/3bcsyk3e

Sincerely,

Eric Baker
Journal Production Manager
MBoC Editorial Office
mbc@ascb.org

Reviewer #1 (Remarks to the Author):

In this revised version the authors have addressed the points of concern raised in my initial review in an appropriate manner. They clarify that the number of WPB is not altered by beta-PIX knockdown, they provide some explanation for the rather low number of WPB fusion events observed, they used additional stimuli elevating cellular cAMP levels and provide some mechanistic ideas on the action of beta-PIX in WPB exocytosis. As such, the revisions have improved the manuscript significantly.

A few points, mainly concerning the new experiments, should be considered:
1. Fig 6B/C. Effect of beta-PIX KD on cofilin Ser3 phosphorylation. The effect at 0 min of PMA stimulation seen in the blot (Fig 6B) doesn't seem to match the quantification (Fig 6C). Did some mix-up occur and why are different grey scales used in 6C?
2. Results, lines 221/222. The authors describe both serine-3 mutants of cofilin (S3A and S3E) as constitutively active. This is somewhat misleading, active in which respect?
3. Fig 7, new schematic. This rather simple model doesn't add much information. Either, it should be expanded to include other factors shown to participate in the process or it should be omitted.

Queen Mary
University of London
Barts & The London

Dr. Tom Nightingale,
Reader in Cell Biology.
Barts & The London
School of Medicine & Dentistry,
Queen Mary University of London,
Charterhouse Square,
London. EC1M 6BQ
Email: t.nightingale@qmul.ac.uk
BSCB Committee member http://bscb.org
Tel: +44 207 882 8238
Fax: +44 207 882 8257

Date 08/04/2026

Dear Editorial board,

Please find attached the revised version of an article submitted to Molecular Biology of the Cell (MBoC) entitled "*Endothelial β-PIX (ARHGEF7) supports actomyosin mediated expulsion of VWF through dynamic reorganization of the cytoskeleton".*

In light of the reviewers comments we have further revised the manuscript. We have changed the gel image shown in Fig. 6B to one that better reflects the average change that occurs in Cofilin phosphorylation. We have also included the experimental data from all 3 experiments as a new supplementary figure (Fig. S4).

We have corrected the error in the description of the S3E cofilin mutant, it is a dominant negative mutant rather than a constitutively active mutant.

We have removed the schematic (previously Fig.7).

We feel the revised manuscript addresses the reviewers remaining points and makes it suitable for publication in MBoC. We do hope therefore that you will consider it.

Kind regards

Tom Nightingale

RE: Manuscript #E25-10-0483RR
TITLE: "Endothelial β-PIX (ARHGEF7) supports actomyosin mediated expulsion of VWF through dynamic reorganization of the cytoskeleton."

Dear Dr. Nightingale:

I am pleased to accept your manuscript for publication in Molecular Biology of the Cell.

Best regards,

Rob Parton
Monitoring Editor
Molecular Biology of the Cell

Dear Dr. Nightingale:

Congratulations on the acceptance of your manuscript! Thank you for publishing your work in Molecular Biology of the Cell (MBoC).

Within 10 days, an unedited PDF of your manuscript will be published on MBoC in Press, an early-release journal version. The date your manuscript appears on this site is the official publication date.

Your copyedited and typeset manuscript will be scheduled for publication in the next available issue of MBoC. Once your paper is ready for review, our production team will notify you. In the summer of 2024, our production provider will introduce an online proofing solution, which will streamline the process of checking and validating author changes. This will result in improved typesetting quality, enhanced digital content, and a faster overall process.

We invite you to submit images related to your accepted manuscript to be considered for the journal cover.
Cover images must contain color, TIF Format
Size image at 7.4" wide x 5.85" deep
RGB and CMYK color (please submit two separate files, one in each format)
300 dpi resolution

Please also send a 1-paragraph description of the image that a general audience could understand. Include with the caption the name and institution of the individual(s) the image should be attributed to. Please provide this description in a Word file. You can send the file via email or by your favorite file transfer service to mboc@ascb.org.

Authors of Articles and Brief Communications are also encouraged to create a short video abstract to accompany their article when it is published. These video abstracts, known as Science Sketches, are up to 2 minutes long and will be published on YouTube and then embedded in the article abstract. Science Sketch Editors on the MBoC Editorial Board will guide you as you prepare your video. Information about how to prepare and submit a video abstract is available at www.molbiolcell.org/science-sketches. Please contact mboc@ascb.org if you are interested in creating a Science Sketch.

We look forward to publishing your paper in MBoC.
Sign up for MBoC's new content alerts here https://tinyurl.com/3bcsyk3e

Sincerely,

Eric Baker
Journal Production Manager
MBoC Editorial Office
mbc@ascb.org
